# Supplementary material for: Asynchronous Antarctic and Greenland ice-volume contributions to the last interglacial sea-level highstand
Source: Nat Commun. 2019 Nov 6;10:5040. doi: 10.1038/s41467-019-12874-3 (PMC6834665; doi:10.1038/s41467-019-12874-3)
Supplement: Supplementary file 1 — Supplementary Information [file 41467_2019_12874_MOESM1_ESM.pdf]

Supplementary Information:

Asynchronous Antarctic and Greenland ice-volume contributions to the last interglacial sea-level highstand

Rohling et al.

Supplementary Note 1.

Stratigraphic evidence of Last Interglacial sea-level instability

The following is a discussion of selected Last Interglacial (LIG) sites that contain stratigraphically coherent records of sea-level oscillations, mostly from far-field locations (*Supplementary Figure* 1). We first discuss sites with stratigraphic superposition (section 1A), followed by sites with reef architecture or geomorphology consistent with intra-LIG sea-level oscillation(s) but where sediments or reef units do not overlie one another directly (section 1B) (for a map of sites discussed, see *Supplementary Figure* 1).

This is intended as an overview (rather than an exhaustive review) of the extensive literature on LIG sea levels. There is much divergence among records, but we provide a short synthesis (section 1C) that portrays an emerging picture of LIG sea levels from coral reef evidence. We report facies and stratigraphic interpretations, and ages as reported in the original publications. In addition, the sea-level archive and key features of the record are given in square brackets, where key features are: mH = multiple LIG sea-level highstands; Fall = inter-LIG sea-level fall(s); Stillstand = LIG sea-level stillstand(s); oscillation = oscillations in LIG sea levels; mPG = multiple phases of LIG reef growth; ? = evidence uncertain or debated.

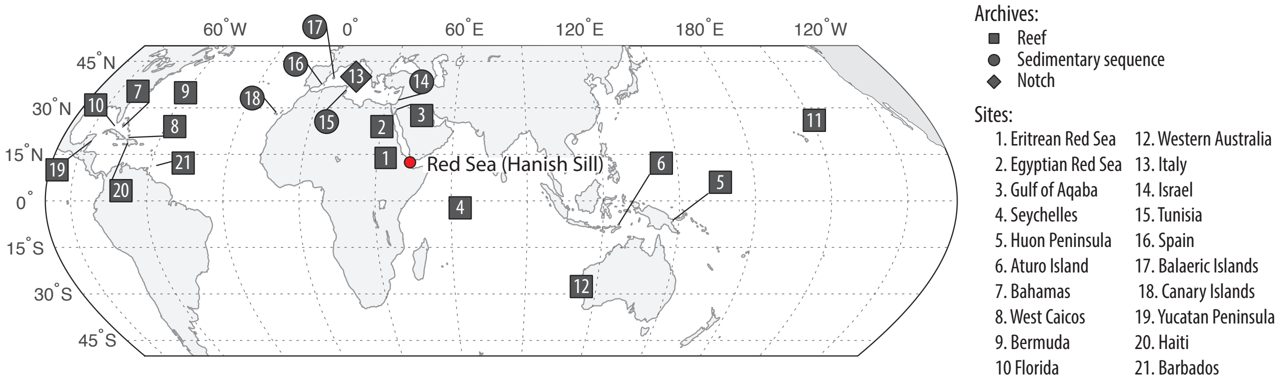

Supplementary Figure 1. Global summary of stratigraphic evidence for Last Interglacial sea-level instability in coral-reef deposits and coastal-sediment sequences. Red dot is the location of the Red Sea sea-level record.

(1A) CONSTRUCTIONAL REEF OR SEDIMENTARY SEQUENCES

1. Red Sea

1.1. Eritrean Red Sea coast [reef; mH, Fall & mPG]:

The Abdur Reef Limestone complex is a MIS 5e marine terrace sequence that contains two superimposed stages of shallow reef development[1,2]. The lower unit is truncated by an intermittent marine erosion surface, which is directly overlain by reef-crest/reef-front coral assemblages. The erosional surfaces that separatethe marine subunits are interpreted as periods of interrupted sedimentation and reef growth[2]. The complete LIG sequence is: a basal lag deposit overlain by grainstone or floatstone facies, which fines upward to suggest rapid deepening. This was followed by shoaling and development of a local reef and the coral proliferation. The top of this marine subunit is a hardground/erosional surface with submarine lithification, and with biological reworking evident. This is inferred to have occurred when the surface was at intertidal depths, during or slightly after the sea-level lowering (the authors are uncertain if the hardground ever emerged). The erosional surface

is overlain by extensive coral reef growth and the typical corals exposed suggest that this is a reef flat in growth position (the authors suggest that this platform rim was near sea level). This reef unit is overlain by reef subunit $5e_3$ with encrusting oysters on the surface that may indicate an additional sea-level lowering or still stand. A subsequent sea level rise is indicated by patches of *in situ* corals that overlie this reef unit[2]. Precise age control for these reefs is difficult; all current U-series dates[1] do not meet commonly accepted age 'reliability' criteria.

1.2 Egyptian coast, Red Sea: [reef; mH Fall & mPG]:
A continuous coastal reef and beach unit extends along nearly 500 km of the Egyptian Red Sea coast[3]. Three distinct sea-level oscillations are suggested for the LIG: (1) a compound first phase with (*1.1*) an initial highstand (elevations ~+6 to +8 m) and (*1.2*) a subsequent (brief) transgression (~+3 m above the previous highstand) - note, this second phase is seen only at the protected sites of Sharm el Naga and Sharm el Bahari, which suggests limited reef growth during this second phase; (2) a short-lived lowstand (with a sea-level drop of up to 10 m[Ref.4,5]; and (3) a subsequent (final) sea-level rise (~+6 m elevation). U-series ages for corals in this region are often affected by diagenesis and open system behaviour. Plaziat et al. (1998)[Ref.4] derive ages for LIG sea-level events by correlating with the global $\delta^{18}O$ stack of Pisias et al. (1984) rather than by direct dating.

1.3 Gulf of Aqaba: [reef; oscillation; mPG]
A flight of coral terraces (on an uplifting coastline) offers potential age constraints for the Red Sea LIG coral record. The superimposed reefs are evidence for sea-level oscillations, with one and possibly two stillstands, during the last interglacial. The three coral units are dated at the "Bedouin Village" site[6,7]. The highest terrace (R3) has limited expression and altered ages (elevation +20 m apsl) but suggests an "earliest part of the MIS 5e highstand around 132-130 ka". The second LIG terrace (R2) (elevation +12 to +18 m apsl) is found a couple of metres below R3 in elevation with sea levels inferred to be +5 m apsl[6]. It should be noted that the corals have been altered extensively to calcite (evident in most corals in the R2, R3 and R4 terraces), which complicates dating of these corals. Ages for these sites are calculated assuming recrystallization during a single period of open-system behaviour that lasted a few hundred years, followed by closed-system behaviour[7].

2. Seychelles: [reef**;** FALL, mPG]
The Seychelles record[8–10] contains evidence of multiple (superimposed) LIG reef growth generations, with at least one short-lived ("ephemeral") sea-level fall/stillstand (evident as distinct lithological and assemblage changes/coral rubble layer). Exposures of patchy veneers of marine limestones that adhere to granitic boulders in an area protected from weathering contain exhibit a conglomerate/rubble layer intercalated between coral units[8–10]. There appear to be "at least three distinct reef-growth episodes punctuated by two discontinuities that typically manifest as coral rubble layers or extensive encrustations of the hydrozoan coral *Millepora exaesa*"[9,10]. At two sites, extensive dissolution and freshwater cements are associated with the disturbance layers, which suggest sub-aerial exposure followed by marine inundation[10]. U-series ages for the rubble deposition event between the first and second episodes of reef growth are regionally consistent between two islands at ~126-125 ka[Ref.9] and "may reflect ephemeral sea-level fall"[Ref.10]. Further, "field evidence and dating from high marine limestones from two sections at La Digue Island indicate a period of coral buildup until 131,000 yr B.P., followed by a drop in sea level between 131,000 and 122,000 yr B.P." (Israelson and Wolfarth, 1999). However, "…only two corals from the

Israelson and Wohlfarth (1999) study pass the screening criteria (Fig. 7a): these two corals have identical ages (123.8 ± 0.5 ka) and occur at an elevation near +4 m. These two samples (90/1 and 90/2) are described as being capped by coralgal-vermetid layers (Israelson and Wohlfarth, 1999), and if they grew up to the intertidal zone may represent a drop in sea level from the +6.6 ± 0.2 m attained at 125.1 ± 0.4 ka."[Ref.9]. Dutton et al. (2015)[Ref.9] could not replicate/corroborate this "tentative" interpretation because the outcrop had subsequently weathered away. However, the most recent study by Vyverberg et al. (2018)[Ref.10] confirms "clear evidence of multiple interruptions in reef growth where well-developed reef units are separated by disturbance… Our observations are consistent with prior suggestions that the LIG sea level highstand was characterized by multiple peaks in sea level". Ages for the two reef-growth episodes described by Vyverberg et al. (2018)[Ref.10] are not yet published. Israelson and Wolfarth (1999)[Ref.8] suggested a magnitude of any sea level fall of ~2 m but, given that reefs are primarily constructional features, this estimate may not fully capture the full range of any sea level fall.

3. Huon Peninsula, Papua New Guinea: [reef; mH, Fall?, mPG]
The Huon Peninsula has an extensive (laterally more than 80 km[Ref.11]) flight of uplifted terraces. The LIG reef VII complex consists of a barrier reef (VIIb), a lagoon, and a fringing reef (VIIa), which has led some to suggest the possibility of two phases of rapid sea-level rise[11–13]. A significant sea-level oscillation is inferred between VIIb and VIIa, based on reef-growth interruption beneath the VIIb barrier (marked by a visible sloping surface in one exposure, with no coral growth crossing the surface[11,12]. Aharon et al. (1980)[Ref.14] described this as an erosional disconformity, associated with a "minor" sea-level fall following an initial sea-level rise and a subsequent < 8 m rise "during the building of VIIb crest"[14]. However, a subsequent expedition in 1988 found no distinctive subaerial features associated with this inferred reef cessation/sea level fall[13]. U-series ages[13] for these two reef units fall into two distinct groups; reef unit VIIb ages cluster at about 118 and 143 ka, and VIIa corals (~3 m below crest of VIIb) centre at about ~118 ka. The apparent lack of corals with intermediate ages led Stein et al. (1993)[Ref.13] to suggest two episodes of LIG sea-level rise, despite significant diagenetic alteration (recrystallization from aragonite to calcite) of corals.

4. Atauro Island: [reef; FALL, mPG]
Atauro Island (north of East Timor/Timor-Leste) has an inter-fingering sequence of reef units, with Reef 2 corresponding to the LIG and an unconformity separating two LIG transgressive subunits[11,12]. In general, three units are recognised in reef 2; reef 2 main (the main body of the reef) overlies an older reef (reef 2-lower); reef 2-main is capped by cobble pavement, which is in turn overlain by a shallow water reef. An additional reef 2-late is recognised in one location and is a small reef remnant beneath a cliff cut into reef 2[Ref.12]. This sequence is interpreted as an initial episode of reef building, followed by a sea-level fall and a subsequent reef-growth episode (reef 2-main). Reef 2-main was possibly interrupted (given the gravels separating reef 2-main and reef 2-upper), although this may have been due to continued tectonic uplift rather than a sea-level change. A major sea-level lowering event (~27 m) may have been interrupted by minor sea-level rise and growth of reef 2-late[Ref.12].

5. Bahamas: [reef; mH; Fall, mPG]
An extensive erosional surface has been described from two islands in the Bahamas (San Salvador and Great Inagua islands)[15–20] and an intra-LIG unconformity has been mapped for 5 km in West Caicos[21] (in the nearby British West Indies, see section 5.1 below). In the

Bahamas, this erosional surface, with shallow-water borings and burrows, separates two LIG reef units. On San Salvador, the Cockburn Town reef erosional surface truncates coral-rubble calcarenite and *in situ* corals. This surface is also encrusted with shallow water borings and burrows and occasionally a palaeosol (red caliche) is preserved[16,17]. On Great Inagua, the Devils Point reef erosion surface extends over several kilometres and again truncates coral-rubble calcarenite and *in situ* corals. The surface has lithophagid and sponge borings with rhizomorphs encrusting the surface. This erosional surface in turn is overlain by LIG corals[15–17]. The initial reef unit formed at ~+4 m above present mean sea level (apmsl) and the intra-LIG event is dated at ~125 to 124 ka and lasted <1.5 ka[Ref.15], followed by a sea-level fall of ~4 m and subsequent sea-level rise of ~+6 m[Refs.15–17]. However, preservation of delicate reef-crest facies[22,23], the apparent lack of *Acropora palmata* reef crests above +3 m, and inter-tidal notches preserved at +6 m[Ref.22] led Hearty and Neumann (2001)[Ref.24] and Neumann and Hearty (1996)[Ref.22] to suggest that the ~+6 m sea-level highstand was of short duration and occurred at the end of the LIG, rather than during an extended episode of elevated sea levels. These authors suggested that the late stage, m-scale sea-level rise was too brief to permit reef development[22]. Blanchon et al. (2009)[Ref.25] noted the similarity in magnitude of the sea-level 'jump' (~3 m) between the Bahamas and Yucatan Peninsula, and suggested that the lack of reef crests at elevations >3 m is due to processes other than sea-level rise. Recent open-system U-series age determinations on *in situ* corals[19] from the superimposed reef units from San Salvador and Great Inagua islands suggest at least one sea-level oscillation during the LIG highstand - "Bahamian geochronology and stratigraphy indicate four resolvable units, supporting the four oscillations in sea level recorded in Red Sea core KL11 (Rohling et al., 2008). There is a 4 ± 1 kyr age difference between Reefs II and I, which are separated by a wave-cut bench, providing definitive evidence of a sea-level oscillation (White et al., 1998; Wilson et al., 1998; Chen et al 1991)"[Ref.19]. However, recent work could not identify these four units, but "found compelling evidence for at least two distinct generations of reef growth, separated by an ephemeral sea-level fall" (Skrivanek et al., 2018)[Ref.20]. Conventional (i.e., closed-system) datings have so far been unable to differentiate between the ages of the two reef units separated by the erosional surface[15], although the youngest closed-system ages for the lower reef unit (Reef I) are ~124.5 to 125 ka[Ref.20].

5.1 West Caicos, British West Indies [reef; mH; Fall, mPG]
An exceptionally preserved exposure of LIG reef sequence (with distinct lower and upper reef units, with ages of ~126.5 and 120.6 ka respectively) has been mapped over ~8.4 km along the west coast of West Caicos island[21]. The lower/early-LIG unit is a fringing reef, with *Acropora palmata* core and coralline algal crust indicating sea levels of ~+4 m at 126.5 ka. An intra-LIG unconformity (mapped over 5 km) with clear evidence for erosion of the lower reef platform, suggests a brief sea-level fall. In places the lower reef unit is truncated by ~3 to 4 m, suggesting that the intra-LIG sea levels fell to ~+1 m or lower. The upper (superimposed) reef unit is capped with foreshore deposits at ~+5 m elevation (unabraded corals that colonised the erosion surface were dated to 120.6 ka). Following this second highstand, progressively downstepping shorelines document falling sea levels.

6. Bermuda: [reef; ?]
The Devonshire Marine Member (aka Rocky Bay Formation) has been correlated with MIS 5e[e.g., 26,27] and is separated from the underlying Belmont Formation by a solution unconformity/soil pipes/reddish soil-like deposits. The age (and hence sea-level interpretation) of the Belmont Formation is contested[e.g., 28,29]. At Grape Bay, the contact

between the Belmont Formation (U-series dated to the penultimate interglacial) and the Devonshire Marine Member is marked by a geosol/soil pipes suggestive of a period of sub-aerial exposure prior to deposition of the LIG Devonshire Marine Member[29]. Hearty and Kindler (1995)[Ref.23], and Hearty (2002)[Ref.30] (reiterated by Hearty et al., 2007[Ref18], and Hearty and Tormey, 2017[Ref.28]) reassigned the Belmont Formation to a member of the Rocky Bay Formation (correlated with MIS 5e[Ref.26]). As a consequence, these authors propose an intra-LIG sea-level fluctuation (i.e., two highstands[23]). However, recent consensus (based on U-series dating of coral fragments) suggests that the Belmont Formation formed during the penultimate interglacial[26,27,29], rather than during the LIG[18,28,30].

7. Florida: [reef; Fall?, mPG]
Within the Key Largo Formation (emerged coral reef-facies limestone with a maximum elevation ~5.5 m above mean high tide) five distinct units are recognised, and are separated by surfaces that indicate subaerial exposure (Q5 correlates to the LIG[e.g., 31]). At Windley Key, Fruijtier et al. (2000)[Ref.32] documented an erosion surface. A coral (sampled at ~+3 m above mean high tide) below this erosion surface was dated to 125 ka. However, this sample has a calculated $\delta^{234}U_{initial}$ value outside of the modern range despite low percentage calcite and ^{232}Th concentration. Diagenetic alteration during the first 40 ka after formation is thought to account for generally older than expected ages at the site[32]. The Miami Limestone (ooid shoals or bars and correlative of the LIG Key Largo Formation) reaches a maximum elevation ~+7.5 m apmsl. An unconformity at ~1.2 m apmls separates two oolitic units[Refs.31,33]. However, an intra-LIG sea-level fluctuation was not recognised from a subsequent stratigraphic and dating study[31].

8. Hawaii: [reef; mH,? Fall?, mPG]
A continuous sequence of strata exposed at Barbers Point, Oahu, includes two stratigraphically distinct highstand units (units II and V) that are separated by "a regressional sequence including *in situ* slabs of beachrock"[Ref.34]. Initial U-series ages of the two marine units suggest a gap of several thousand years between deposition of these two layers[34]. Elsewhere on Oahu, the top of the Waimanalo Formation (U-series dated to the LIG [e.g., 35,36]), an *in situ* coral-algal framestone, is often planar (e.g., Costa Dairy, now quarried away[18,37]) and in some instances erosionally truncated on its upper surface. This "erosional unconformity" represents the mid MIS 5e lowstand and separates the framestone from overlying grainstone and rudstone (Leahi Formation) that accumulated during the second 5e highstand)[34]. Mokapu Point (east coast of Oahu) contains two stacked *in situ* coral reefs, separated by a terrigenous basalt conglomerate, with no evidence of subaerial exposure. Similarly, the Kahe Beach State Park sequence includes two exposed *in situ* "reef levels" capped by marine conglomerate[18].

Amino acid racemization (AAR) dating of the Waimanalo Formation[38] appears to confirm the age separation of the two marine units[cf. 34] and was used as further evidence for two sea-level highstands separated by a minor regression. Subsequent U-series dating of the various LIG units confirm a LIG age, but unit ages are largely indistinguishable from one another[18,29,35,36,39] and "fail to corroborate the exquisite lithostratigraphic succession of this site, as most ages do not pass reliability standards"[Ref.18], yet Muhs et al. (2002)[Ref.29] "do not see any persuasive evidence for two separate high stands… as interpreted by Sherman et al. (1993) from elsewhere on Oahu".

9. Western Australia: [reef; Fall, mPG]
(see also section 12 for discussion of LIG coral evidence)
A sharp unconformity (erosional surface/abrasion platform formed within a LIG rocky shore) has been documented within the Tamala Limestone Formation (Cape Burney, near Geraldton)[40]. This low-relief, channelled surface formed on calcareous sandstone and is encrusted by intertidal to shallow subtidal biota (coralline algae, serpulid worms) and coral. It is overlain by a reef unit dominated mostly by undisturbed coral fronds of robust *Acropora* species[40]. However, the age and sequence of events, particularly the late LIG highstand in Western Australia, remain controversial.

10. Mediterranean
Multiple sites within the Mediterranean are inferred to contain evidence of LIG sea-level oscillations. A thorough review of this literature is beyond our scope, and only a brief discussion of key sites is given. The Mediterranean has a complex tectonic setting but small tidal amplitude and low wave energy. Evidence of former sea levels comes from a range of sea-level archives - depositional (beach or shallow marine deposits[e.g.,41–43], geomorphological (shore platforms or notches) or fixed biological indicators[e.g., 44]. Dating of deposits is challenging because there are few corals for U-series dating and this technique is unreliable when applied to fossil molluscs[e.g., 45]. We do not reinterpret or recalculate ages from the original publications and instead concentrate on the stratigraphic evidence for potential sea-level oscillations. Due to the problems of obtaining reliable age control for Mediterranean Quaternary sediments, fossil mollusc assemblages (e.g., the warm "Senegalese fauna"[e.g., 46]) are often used to identify LIG deposits (due to their temperature sensitivity), with *Strombus bubonius* (*Persististrombus latus*), which is extant in the tropical waters off west Africa but not in the Mediterranean today, particularly diagnostic[e.g.,45]. It should be noted that some authors[e.g., 43,46] suggest that this fauna is neither synchronous nor continuous throughout the Mediterranean during MIS 5e[Ref.46] and it has also been found in older interglacial deposits[e.g., 43], although these conclusions are based on U-series dating of fossil molluscs[45].

The complex Mediterranean tectonics, coupled with dating uncertainty, has made deconvolution of LIG sea-level history of the basin difficult. For example, only one highstand is recognised in the "generally stable" tectonic setting of Sardinia based on tidal notches with a mean elevation of +6 ± 3 m (apmsl) (Ferranti et al. (2006)[Ref.47] and references therein), and from shoreline evidence from multiple Mediterranean sites[42,48], whereas multiple highstands are inferred for sedimentary sequences elsewhere in the basin[e.g.,43,46,49,50].

10.1 Italy (including Sardinia & Sicily): [notches; mH?, oscillations?]
Emerged tidal notches, including "double notches" (tidal notch couplets) or superimposed bioerosional grooves, are preserved at many sites in Italy due to the microtidal regime[44,47,51]. The upper notch is commonly attributed to MIS 5e and the lower to later stages within MIS 5, although firm age control remains elusive. Superimposed bioerosional grooves associated with upper notches at ~+5 m elevation in the Gulf of Orosei and Bergeggi Marine Cave (+5.24 m, +4.40 m, +3.52 m and +2.7 m elevation) are thought to have formed during distinct highstands within MIS 5e [Ref.44]. However, Antonioli et al. (2006)[Ref.51] argue that both notches of the tidal notch couplets formed during the LIG, with the lower notch forming during the earlier portion of the LIG, although this was attributed to glacio-isostatic adjustment (GIA) processes, rather than sea-level fluctuations and hence the different morphology of the two notches within the couplet[51]

10.2 Israel (Galilee coast): [sedimentary sequence; mH; stillstand/Fall]
The Rosh Hanika site is a micro-tidal, tectonically stable location that contains a complete stratigraphic sequence for MIS 5e, although U-series ages from molluscs are altered (open-system)[50]. The continuous shore sequence is as follows (generalised from sites in the wider region, with only the Rosh Hanika site containing the complete stratigraphic section): first comes the the Regba Member, a calcareous sandstone (aeolian dune, tentatively ascribed to MIS 6, with upper planar beds characteristic of a shallow marine or coastal environment). In some locations (e.g., Hazrot Yasaf) abrasion platforms are evident (at +2.6 m and +3.4 m apsl), which were cut by tidal channels. The authors suggest that these were cut during relatively long sea-level stillstands as part of two sea-level-rise steps within an initial MIS 5e sea-level rise (see note below *). The Regba Member is overlain unconformably (interpreted as a sea-level drop) by the Yasaf Member, which contains a gravel unit with *Strombus bubonius* fossils (warm water fauna used as a marker of MIS 5e in the Mediterranean). This is in turn truncated by an unconformity, which is inferred to have been caused by a sea-level fall that caused a relatively short period of emergence, and which is in turn overlain by a *Vermetidae* reef (indicative of a low energy environment) capped by algal crust (inferred shallow water deposition). There is another unconformity (cessation of reef formation, likely due to sea-level lowering), that is overlain by two bioclastic sandstone subunits (subsequent transgression). After this, sea level dropped and the coastline retreated offshore[50].

* Abrasive notches exposed along the Galilee coast suggest sea levels slightly higher than present at the start of MIS 5e, with an upper limit between +0.5 to +0.75 m. The notches contain two subunits of the Yasaf Member, which indicate relatively long stillstands at an elevation of ~+1 m, and that the early MIS 5e erosive phase was followed by a depositional phase later in MIS 5e[Ref.49].

10.3 Tunisia: [sedimentary sequence; mH?, Fall?]
The Hergla site in Tunisia contains a facies succession that includes two foreshore deposits, each overlying a possible erosion surface[18,52,53]. The lower unit is a siliciclastic unit devoid of warm water fauna (~+2 to +3 m apmsl, aged 147 to 110 ka from U-series dating of molluscs) capped by aeolian sediments that are overlain by a carbonate-rich, shallowing-upward marine unit that contains warm marine fauna including *Strombus bubonius* (ages derived from U-series dating of *Ostrea lamellosa* shells range from 141 to 100 ka), capped by a *Strombus*-rich boulder bed (elevation ~+3 to +6 m apmsl)[52]. These units are interpreted as two MIS 5e highstand deposits that developed during two sedimentation phases, during two distinct sea-level highstands based on sedimentology, faunal assemblages and U-series dating of molluscs/a coral, and amino-acid dating[18,41,52,54]. Hearty et al. (2007)[Ref.18] interpreted the top of the aeolian deposit (capping the first unit) to be a weathering surface associated with a sea-level fall "to near or below present", whereas Mauz et al. (2018)[Ref.53] relate this to lagoonal sediments, which suggests shoreline migration. Recent OSL dating of the lower (110 and 120 ka) and upper units places the sea-level rise associated with formation of the second package in MIS 5a, rather than a second LIG highstand[Ref. 53].

10.4 Spain: [sedimentary sequence; mH; Fall]
A variable number of highstands associated with MIS 5e is recognised on the Spanish coast[e.g., 55], with the greatest number documented on the Mediterranean coast (primarily due to tectonic uplift)[e.g., 56]. Three LIG highstands for the Iberian Peninsula have been inferred based on extensive geomorphological mapping, dating and facies analysis[e.g.,43,46,57]. The general sequence is: (1) a first LIG highstand (characterised by oolitic dunes and beaches containing

Strombus bubonius), (2) a second highstand with the highest elevations and containing "two morphosedimentary subunits separated by an erosional surface"; and (3) a brief third highstand in which sea level was slightly lower[55]. For example, the Loma del Viento section[43,58,59] contains a laterally extensive 'staircase' of marine units, four of which contain *Strombus bubonius.* These are terraces 12, 13, and 14 with present elevations +14, +6, and +3 m apmsl, respectively (following the stratigraphic subdivisions of Zazo et al. (2003)[Ref. 43]) and three LIG sea-level oscillations are proposed based on U-series and AAR dating[58–60]. Similarly, the El Pinet site (an abandoned quarry) contains five prograding units (numbered 7.1 to 7.5 in Zazo et al. (2003)[Ref.43]), all containing the warm "Senegalese" fauna and *Strombus bubonius*. Unit 7.1 (sepulid/bioclastic limestone with patches of encrusting coral indicative of shallow marine environments, assigned to MIS 7) is overlain by an oolitic calcarenite. An erosional layer separates unit 7.2 and overlying siliclastic sandstones and conglomerates (unit 7.3). Erosional layers also separate LIG unit 7.3 and overlying units 7.4 (calcarenite) and 7.5 (calcarenite, sandstones and conglomerates which is the "richest" in *S. bubonius*)[43].

10.5 Balearic Islands – Mallorca: [sedimentary sequence; mH?, Fall?]
Emergent marine deposits (elevations of +2 and +3 m apmsl) that are dated (or inferred) to be of MIS 5e origin are documented from several locations on Mallorca[41,43,46,48,57]. Two (and possibly three) distinct sea-level highstands are proposed during the LIG, one early at ~135 ka and two at ~117 ka[Refs.41,43,46,57], although these ages (except Hearty, 1986[Ref.41]) are based on potentially unreliable U-series mollusc dating. The ages and elevations of the marine deposits correspond to speleothem (phreatic) overgrowths from coastal caves at elevations of +1.5 m to +2.6 m dating from ~138 to 110 ka[Refs.61–63]. Tuccimei et al. (2007)[Ref.64] proposed that two episodes of speleothem growth are separated by a rapid LIG regression/lowstand at ~125 ka. At the Campo de Tiro site, marine units (~0 to +3 m elevation apmsl) are separated by reddish terrestrial deposits or erosional surfaces[41,43,57]. However, the precise age and number of LIG sea-level oscillations (highstands) at this site are debated. Hearty (1986)[Ref.41] recognised three marine units, whereas four marine units were documented by Bardají et al. (2009)[Ref.46], Hillaire-Marcel et al. (1996)[Ref.57] and Zazo et al. (2003)[Ref.43].

A marine unit (unit 2 of Zazo et al. (2003)[Ref.43]); max elevation ~+3 m[Refs. 43,48]) is underlain by a thick red silt layer. Note that Zazo et al. (2003)[Ref.43] also assign the marine unit below this silt layer (elevation +1.5 m apmsl) (unit 1) to the LIG due to the occurrence of warm-water fauna, including *Strombus bubonius*, in both units, whereas Muhs et al. (2015)[Ref.48] attributed the aeolianite, from which the palaeosol developed, as likely formed during MIS 6. The third marine unit of Zazo et al. (2003)[Ref.43] and Bardají et al. (2009)[Ref.46] (unit 3, elevation of +1 m apmsl), also contains warm-water fauna but without *S. bubonius*, overlies an erosional surface that truncates both units 1 and 2. However, this unit was not recognised in the later fieldwork of Muhs et al. (2015)[Ref.48]. In contrast, Muhs et al. (2015)[Ref.48] documented seaward "Neotyrrhenian" beds (max. elevation +2 m apmsl) that consist of a lower layer of gravelly sands (with few fossils) and an upper sandy gravel layer containing abundant fossils. These beds overlie a reddish-brown palaeosol, which was found to be a aeolianite, which is in turn overlain by marine deposits documented at +3 m. These seaward "Neotyrrhenian" beds were interpreted as a beachrock facies that formed later during the same highstand as the +3 m marine deposits[48].

U-series dating of molluscs and stratigraphic evidence led Hillaire-Marcel et al. (1996)[Ref.57] and Zazo et al., (2003)[Ref.43] to suggest a MIS 5e origin for all three marine units (units 1, 2, and 3), in which the youngest was assigned to a separate, later LIG highstand based on facies

and faunal considerations[43]. U-series dating of fossil corals and amino-acid dating of molluscs from the uppermost portion of the "Neotyrrhenian" beds suggest an age of ~120 to ~123 ka for this deposit[41,48], but Muhs et al. (2015)[Ref.48] consigned all the marine units to the same highstand despite the different sedimentology of the two marine deposits. Glacio-isostatic processes were invoked to account for the two marine units by Muhs et al. (2015)[Ref.48], given the small altitudinal separation (~1 m) between the documented marine units.

10.6 Canary Islands: [sedimentary sequence; mH, Fall]
Marine deposits containing *Strombus bubonius* have been documented at low (<12 m) elevations on many Canary Islands[43,65], but robust age control is lacking. On Lanzarote (El Berrugo), three stratigraphically superimposed MIS 5e units with a sharp erosional surface between units 2 (calcarenite containing *Strombus bubonius*) and 3 (cemented conglomerate including pebbles eroded from earlier units, interpreted as a beach deposit) were used to suggest subaerial exposure prior to deposition of unit 3 during the LIG, possibly indicating two highstands[43]. On Fuerteventura Island, the stratigraphic section at Rosa J. Sánchez site contains alternating marine (3 units) and terrestrial (two) units, with U-series dating of mollusc shells suggesting MIS 5e ages for all marine units[43]. The Playa de Igueste site (Tenerife) comprises two superimposed marine units; the lowermost marine (conglomerate) unit contains *Strombus bubonius* and is separated from the upper marine unit (beach conglomerate also containing *Strombus bubonius*) by a terrestrial silty deposit, which suggests the presence of two MIS 5e highstands interrupted by a possible period of sea-level lowering[43].

(1B) REEF ARCHITECTURE

11. Yucatan, Mexico: [reef; oscillation - sea-level "jump", mPG]
A laterally extensive back-stepping LIG reef sequence has been documented[25,66] from a tectonically stable site. The complete reef sequence consists of "two separate linear reef tracts with reef crests that are offset and at different elevations"[Ref.25]. This backstepping sequence was used by Blanchon et al. (2009)[Ref.25] to suggest sea-level instability (a sea-level "jump") during the later stages of MIS 5e at rates similar to those in the Caribbean during the last deglaciation (~ 36 mm/yr)[Refs.67,68]. Currently, the reefs lack good age control, but biofacies and stratigraphic evidence suggest that both reef units are contemporaneous and that the lower unit if older, died suddenly but remained submerged while the upper reef unit back-stepped during sea-level rise, i.e., "reef development during the highstand was punctuated by reef-crest demise at +3 m and back-stepping to +6 m. The abrupt demise of the lower reef crest, but continuous accretion between the lower-lagoonal unit and the upper-reef crest, allows us to infer that this backstepping occurred on an ecological timescale and was triggered by a 2-3 m jump in sea level" [Ref.25].

12. Western Australia: [reef, mH, stillstand, mPG]
A well-developed MIS 5e terrace is documented at ~+2 to +4 m elevation (apmsl) along extensive portions of the Western Australian coastline [69–75]. At Cape Cuvier, two "geomorphologically distinct" MIS 5e marine highstand units—a lower erosional fringing reef (shore platform, formed by wave abrasion in middle to upper intertidal zone elevations at ~+2 m to ~+3.6 m apmsl) and an upper, narrow "underdeveloped" constructional reef at +8 to +10 m apmsl—were used to argue for an extended sea-level stillstand followed by a short-lived excursion of elevated sea level at the end of LIG, reaching perhaps +8.2 m (or even +9.4 m) apmsl late in MIS 5e[Refs.72,74]. In the Shark Bay area, a possible sea-level

regression was suggested given an apparent 'age gap' and inferred abrupt halt in coral growth at ~124 ka[Ref.73]. However, the ages and sequence of events, particularly for the late LIG highstand in Western Australia, remain controversial. Many U-series ages for the LIG in the Australian region suggest pervasive open system behaviour and/or variable diagenesis[e.g., 70,71]. In addition, the +5 to +6 m emergent shoreline mapped at Quobba Ridge (inferred palaeo sea level of +9 m after GIA correction[74]) and the Cape Cuvier upper terrace/rim[72,74] are thought to result from significant neotectonic deformation rather than sea-level fluctuations[76].

13. Haiti: [reef; mPG]
Dumas et al. (2006)[Ref.77] mapped two LIG terraces (T3a and T3b), separated by ~2 m in elevation from a tectonically uplifted terrace sequence[77,78]. U-series dating for the lower terrace gave an age of ~130 ka (inferred relative sea level +5 m apmsl), with the upper terrace dated to ~118 ka (sea level + 2.7 m apmsl). These two sub-terraces are not always distinguishable, and the localised expression is thought to relate to the higher uplift rate at the site surveyed by Dumas et al. (2006) compared to other surveyed sections in the area[78], where the two terraces merge into each other[77].

14. Barbados: [reef; mH; mPG]
The Rendezvous Hill terrace is an emerged LIG reef complex that retains much of its original depositional morphology. Stratigraphic evidence for LIG sea-level instability from the fossil reef is equivocal but some authors have proposed multiple sea-level peaks based on morphology, facies information and dating[18,79,80].

Based on reef morphology and ESR dating, three episodes of constructional reef-terrace formation during the LIG have been proposed[81]. Terrace dating suggests that terrace T5a (~128 ka) and terrace T5b (~ 132 ka) formed during an initial LIG highstand, whereas terrace T4 formed at ~ 118 ka when sea level was several metres below present (ages as originally reported). However, a younger age for the two higher terraces (terraces T5a and T5b) and an older age for the lower T4 terrace (also known as the Maxwell terrace) were obtained using whole-rock amino acid dating[18], which led these authors to correlate the lower T4 terrace with an initial LIG highstand, and the T5 units to a subsequent, higher sea-level highstand during the LIG.

A multi-stage LIG reef development was also suggested from reef-front-architecture variations and facies relationships[79]. Using a detailed facies approach, these authors suggest that a brief episode of rapid sea-level fall and possibly a minor stillstand led to the reef development at ~16 m below the original reef crest (cf. Maxwell terrace/T4 terrace of Schellmann and Radtke, 2004). However, lack of duplicate reef architecture suggested that any LIG oscillations must have been rapid (hence the lack of a constructional reef record)[Ref.79]. A sea cave at +6 m is thought to have been cut during the LIG, and was used to infer reef-growth cessation prior to the peak (maximum) sea level, possibly due to a change in environmental conditions or a jump in the rate of sea-level rise (rate of rise > rate of reef accretion)[Ref.79].

(1C) CHALLENGES OF REEF STRATIGRAPHY
Reef accretion is complex and results from an interplay of many factors that includes physio-chemical parameters (irradiance, temperature, hydrodynamic energy etc.), the composition of reef communities and their potential rates of growth/bioerosion, balance between

sedimentation vs. calcification, reef disturbance (storms etc.), and variations in coral recruitment, as well as the rate and amplitude of sea-level change (for further discussion, see the reviews of Scoffin *et al.*, 1980[ref.82], Montaggioni, 2005[ref.83], Hubbard, 2009[ref.84], Woodroffe and Webster, 2014[ref.85], Camoin and Webster, 2015[ref.86], Hibbert *et al.,* 2016[ref.87]). In addition, taphonomic and diagenetic processes, and potential coring artefacts, have implications for interpreting spatial variation and the rates and style of framework development[88–92]. For example, coral skeletons are frequently reworked in many reef settings, with selective destruction of certain growth forms, individuals, and age-classes, as well as a mixing of successive generations (also known as time averaging – both ecological and sedimentological[e.g., 88,93,94]). Reworking by storms/hurricanes etc. can also exert a strong control on reef anatomy, such that *in situ* framework is lacking, and instead the reef consists of coral-cobble rudstone layers (e.g., Blanchon et al., 1997[ref.95]). The latter led Hubbard et al. (1990[ref.96]) to state that, for many reefs in the Caribbean, "…the importance of detrital material in the reef fabric and the major role played by secondary processes that constantly rework the substrate have resulted in a reef whose interior is more of a "garbage pile" than an in-place assemblage of corals cemented together into a rigid framework."

Given the interplay of some or all of the above-listed processes, complex age structure is possible and is an important limit on the temporal precision achievable from reef-based sea-level reconstructions[97]. Individual dates from a reef unit that represents a certain time-interval may be stratigraphically jumbled within the unit. Such complex age structures have been reported, for example, for Holocene growth on the Great Barrier Reef[98,99]) and Papua New Guinea (Huon Penninsula[97]).

(1D) SYNTHESIS

The nature of LIG sea-level variability remains strongly debated[e.g.,18,100]. Different models of LIG sea level have been proposed from coral records. These include:

a) relatively stable sea level (i.e., one major peak) (e.g., Stirling et al., 1998[ref.71]);
b) two peaks separated by a sea level fall of various magnitudes (e.g., Chen et al., 1991[ref.15], Stein et al., 1993[13], Sherman et al., 1993[ref.34], Plaziat et al., 1998[ref.4], Bruggemann et al., 2004[2], Thompson and Goldstein 2005[ref.80], Hearty et al 2007[ref.18], Kerans et al., 2019[ref.21]);
c) relatively stable (possibly with a small drop) sea level with a rapid late rise (e.g., Neumann and Hearty, 1996[ref.22], Hearty, 2002[ref.30], O'Leary et al., 2013[74], Blanchon et al., 2009[ref.25]) and;
d) multiple peaks (e.g., Thompson et al., 2011[ref.19], Rohling et al., 2008[ref.101], this work).

The intensively studied, sampled, and dated LIG coral/reef records of the Seychelles[8–10], Bahamas[15,19], and Western Australia[18,70–74] give an emerging picture of LIG sea level that have similarities with the Red Sea record. These coral records are especially useful given that: (1) they span extended periods of the LIG, (2) they have relatively high temporal sampling and density of radiometric dating, (3) they are from tectonically stable areas; (4) they have well-documented stratigraphic superposition of LIG units, and (5) for the Seychelles, there are well-constrained palaeo-water depth estimates. We do not view these records in isolation, but within the well-documented context of the records extensively discussed in sections 1A and 1B.

The GIA corrected Seychelles corals document an initial gradual eustatic sea level rise from ~+5.9 ± 1.7 m to +7.6 ± 1.7 m between 129 and 125 ka, with a possible drop before 125 ka.

A single encrusting coral at +9.2 m at ~125 ka from Camp Rock, Cape Cuvier in Western Australia suggests "a rapid 3 m rise … and fall in sea level at this time"[Ref.74]. Both the Bahamas and Seychelles suggest sea level at ~4 m at ~123 and possibly 124 ka[Refs.9,19] with a decrease (drop) in sea level to ~0 m between 123 and 119 ka in the Bahamas[19]. Sea level highstands at 119.2 ± 0.5 ka (about 6 m), 117.5 ± 0.4 ka, and 114.4 ± 1.0 ka are seen in the Bahamas record as four distinct stratigraphic units, and the possibility of a sea-level drop between each highstand cannot be discounted[19]. A final (and somewhat contentious, given the tectonic setting and potential open-system behaviour) sea-level high of +3.4 m (GIA corrected) at ~118.1 ± 1.4 ka in Western Australia[74] may correlate with either the 119 ka or the 117 ka Bahama deposits[19]. The Barbados coral record[102] is often used to constrain the 'age' of the MIS 5e/d transition. In this study, the ages and elevations of two corals OC4 and OC-1 (dated in triplicate and each satisfying age reliability criteria) bracket the sea-level fall at the end of the LIG. The youngest coral gives a youngest age constraint for the LIG-end at ~113 ka. Yet, in tectonically stable locations, no LIG corals are found that are younger than: (1) ~114 ka in Florida[31]; (2) ~118 ka in the Bahamas[19]; and (3) ~ 117 ka in Yucatan Peninsula[25]. Similarly, speleothem growth began (as a result of sea level fall) at ~ 116 ka in Mallorca[61,62] and was below -4.9 m at 117 ka in Yucatan Peninsula[103].

Supplementary Note 2.

LIG age adjustment in the Red Sea sea-level record

Here we show details of the LIG age adjustment, which is primarily driven by comparison of the overall Red Sea stack record with the Yucatan speleothem-based indications of when sea level first dipped below 0 m again[103], and secondarily by indications from coral-data compilations of when the LIG ended[102,104]. The Red Sea stack is shown on its previous age model in *Supplementary Figure 2* (left). This age model was found to be deficient for the end of the LIG, and comparison with the aforementioned benchmark records reveals that the LIG end is better placed at ~118.5 ka, noting the generous 2σ (95%) uncertainty of ±1.2 ka that applies to the Red Sea age model (*Supplementary Figure 2* bottom; *Supplementary Figure 3*). To make this adjustment, and evaluate its uncertainties, GIA impacts were considered (*Supplementary Figure 4*).

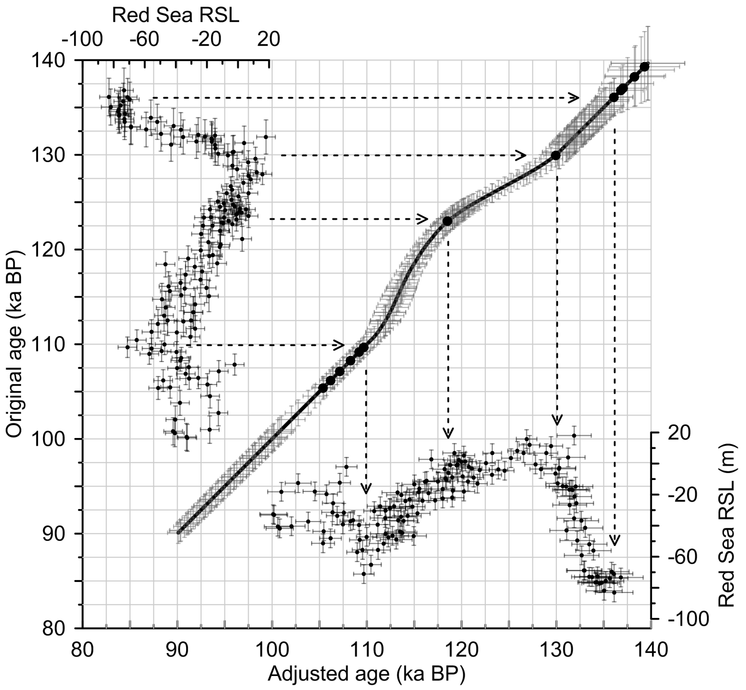

Supplementary Figure 2. Interpolation and propagation of age uncertainties for the adjusted Red Sea Last Interglacial chronology. Elements shown include: the Red Sea sea-level stack on its first radiometrically-controlled chronology[105,106] (left); the adjusted age-control point (central dot at 123 ka on the Y axis, which becomes 118.5 ka on the X-axis, as per adjustment of the 95% upper limit to the coral- and speleothem-based end of the LIG highstand at 118.5 ka; see Figure 2); a number of forced age-control points to effect exact agreement between the chronologies >130 ka and <110 ka (other dots on the 45° line – this ensures that, outside the LIG adjustment interval, the new chronology is identical to the original chronology); and the Red Sea sea-level stack on its adjusted chronology (bottom). Interpolation and uncertainty propagation for the adjusted chronology is described in *Methods.* Arrows visualise the adjustment pathway.

For this analysis, we corrected both the Red Sea record and the Yucatan Peninsula speleothem record for GIA processes using configurations of the penultimate glacial (MIS 6) ice sheets after Rohling *et al.* (2017)[ref.107]. These use a smaller Laurentide Ice Sheet with either: (i) a Eurasian Ice Sheet (EIS) with greater mass but LGM-like spatial configuration; or (ii) an EIS with both greater mass and spatial extent. Fuller details of the chosen Earth model and ice models are given below in *SI Part 4*, where it is shown that the GIA corrections themselves have uncertainties up to ±3 m at the end of the LIG. The exercise used here for evaluating the Red Sea versus Yucatan record after GIA correction uses an artificially defined Global Mean Sea Level (ice-volume) history. Results show that the Yucatan record closely tracks GMSL (*Supplementary Figure 4*). The Yucatan data indicate that Yucatan RSL (and thus by close approximation GMSL) first reach −4.9 m just after ~118 ka (*Supplementary Figure 3*). The upper 95 % confidence bound for Red Sea RSL would sit some 3 m above that (*Supplementary Figure 4*), with an uncertainty up to ± 3 m (*Supplementary Figure 5*); hence our selection of the ~118.5 ka age for the upper 95% bound of the Red Sea record to fall through 0 m. Bearing in mind the generous 2σ (95%) uncertainty of ±1.2 ka that applies to

the Red Sea age model (*Supplementary Figure 2*), this selection of ~118.5 ka is coherent with both the Yucatan data[103] and the Cutler et al. (2003)[102] and Hibbert et al. (2016)[83] coral-based assessments for the LIG end.

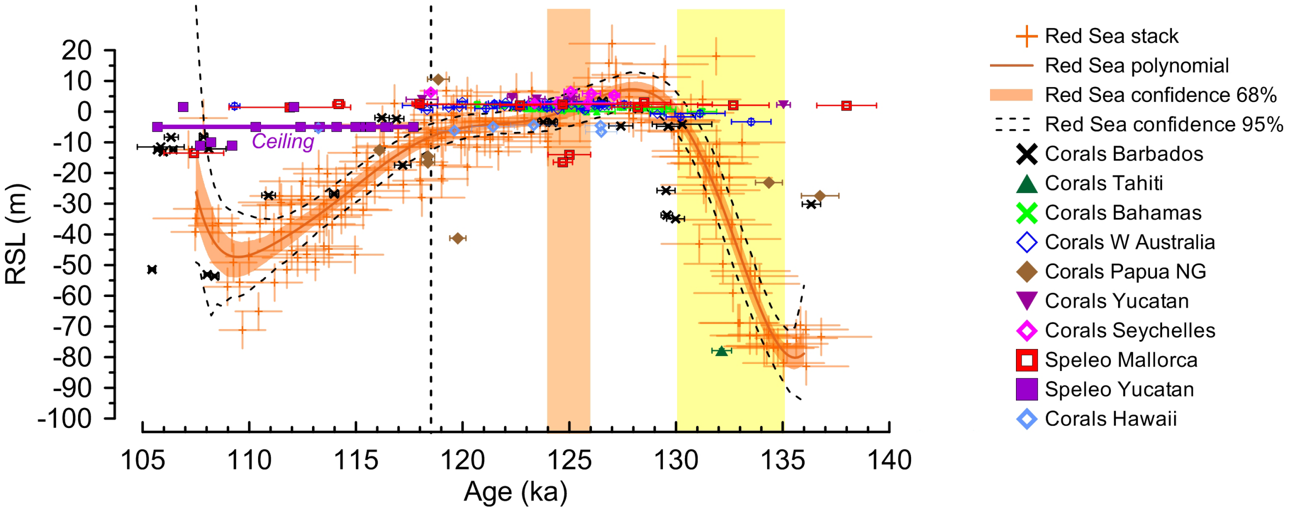

Supplementary Figure 3. Red Sea, coral and speleothem sea-level data. Red Sea stack of relative sea-level (RSL) data with 1σ error bars, polynomial smoothing and 68% and 95% confidence intervals. Coral data, from the compilation of Hibbert *et al.* (2016)[Ref. 87] and Dutton *et al.* (2015)[9], are reported in ka BP, where all coral ages were recalculated (when necessary) using the Cheng *et al.* (2013)[Ref.108] decay constants for ^{234}U and ^{230}Th and assuming closed system behaviour. Corals have been screened for age 'reliability' using the following criteria: calcite <2%, ^{232}Th concentrations <2 ppb, and a calculated $\delta^{234}U_{initial}$ in the range of modern corals (i.e., $\delta^{234}U_{initial}$ = 147 ± 5 ‰). Replicate ages passing the screening criteria are have been averaged (using an inverse weighted mean). Corals are from: the Bahamas[15,19] (green cross); Barbados[79,109–112] (black cross); Tahiti[113] (dark green filled upward pointing triangle); Yucatan Peninsula[25] (purple filled downward pointing triangle); Seychelles[8,9] (pink open diamond); Hawaii[29] (blue open diamond); and Western Australia[69–71,73,75,114] (dark blue open diamond). The Seychelles point at 118 ka is an inverse weighted mean of samples SY-22c (112.2 ± 0.61, 2σ, including decay constant error) and SY22a (124.3 ± 0.56 ka, 2σ, including decay constant error) from site 4 in Dutton *et al.* (2015)[ref.9] (their Table 3, SY-22a 124.5 ± 0.5ka; SY-22b 96.1 ± 0.4 ka; SY-22c 112.4 ± 0.6 ka). Although Dutton *et al.* (2015)[ref.9] remove SY-22a from subsequent analysis as they suspect U-addition, both the SY-22a and SY-22c replicate samples pass our screening criteria, so that we have no objective criterion to eliminate one or the other. We simply plot the mean, but flag a potential issue. Speleothem evidence of past sea levels from phreatic overgrowths from Mallorca[61,62,115] (red open squares) and subaerially deposited speleothems from the Yucatan Peninsula, Mexico[103] (purple filled squares, subaerial growth indicated by solid purple line marked 'ceiling'). Yellow bar denotes the time-interval of Heinrich Stadial 11 (HS11)[116]; orange bar indicates the time-window of potential short-lived sea level lowering observed in, e.g., the Seychelles[8,9] and Red Sea[3,this study]. The relationship between the initial (orange) polynomial assessment of the entire Red Sea stack and the more precise probabilistic assessment of core KL11 alone is illustrated in Figure 2.

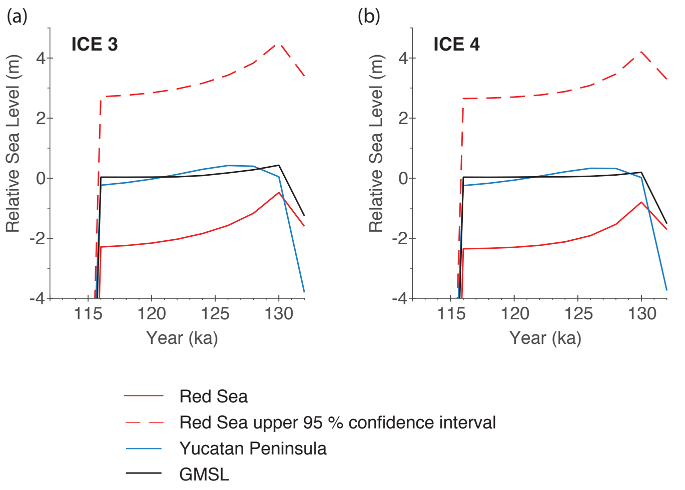

Supplementary Figure 4. Glacio-isostatic adjustment modelling of Yucatan Peninsula speleothem and Red Sea record. GIA predictions of relative sea level for the Red Sea stack (solid red line = median; dashed red line = upper 95 % confidence interval), Yucatan Peninsula (blue) and global mean sea level (GMSL, black) using 'more realistic' MIS 6 ice histories. We use a VM-2-like earth model, a smaller volume Laurentide Ice Sheet and: (a) greater volume Eurasian Ice Sheet with LGM-like spatial configuration (ICE 3); and (b) greater volume Eurasian Ice Sheet with more extensive spatial extent (ICE 4).

Supplementary Note 3.

Greenland mass loss estimates from East Greenland Current sea-water $\delta^{18}O$

Sediment core MD03-2664 (57°26.34'N, 48°36.35'W; 3,440 m water depth) from Eirik Drift[117,118], off the southern tip of Greenland, lies under a system of surface currents that carry most of the melt contribution from the Greenland ice sheet, most notably the East Greenland Current and the wider Labrador Sea systems. Today, Greenland meltwater affects the net seawater $\delta^{18}O$ by addition of water with salinity (S) of 0, and $\delta^{18}O$ of around –30‰. Other (almost) freshwater components, with typical property values are sea-ice melt (typically S = 3, $\delta^{18}O$ equal to ambient water plus 2.1‰), and meteoric water from precipitation and river input (S = 0, $\delta^{18}O$ **=** **–**18‰). These mix with ocean water advected to high latitudes, with typical values of S = 35 and $\delta^{18}O$ = 0.3‰[Refs. 119,120]). It is common practice to use these, or similar, parameters (and where needed also additional water-based hydrogen isotope data[119]) in straightforward end-member mass-balance calculations[e.g., 119–122]. We use such a calculation to consider the amount of Greenland melt-water addition (and, thus, ice-sheet mass loss) needed to cause a –1.3‰ amplitude change in seawater $\delta^{18}O$ at Eirik Drift, as found in core MD03-2664. This amount is equal to the difference between the fraction of Greenland melt before (f_{G0}) and after (f_{G1}) the change. All other terms are kept constant, to enable comparison of the effects due to Greenland melt-water change. We then find:

$$f_{G0} = \frac{\delta_{EGC} - [f_M\delta_M + f_S(\delta_{EGC} + 2.1) + f_A\delta_A]}{\delta_G}$$

$$f_{G1} = \frac{(\delta_{EGC} - 1.3) - [f_M\delta_M + f_S(\delta_{EGC} - 1.3 + 2.1) + f_A\delta_A]}{\delta_G}$$

Here, f is the mixing fraction, δ is the component-water $\delta^{18}O$, EGC indicates the East Greenland Current, M is for meteoric water, S indicates sea ice, A is for advected ocean water, and G is for Greenland melt water. We set the calculation up with modern values f_M = 0.005, f_S = 0.028, f_A = 0.93, and δ_{EGC} = –1‰ (Cox, 2010[Ref.119], p.98). As mentioned above, we kept these values constant in both cases. We thus find that f_{G0} = 0.041, while f_{G1} = 0.083. Using a salinity mass balance and a 5 × 10^6 m^3 per second mass flux of the EGC to calculate mass fluxes, the change in mixing fraction then implies 1.311 × 10^{-3} m per year of additional global sea-level addition due to Greenland melt-water input for the full –1.3‰ seawater $\delta^{18}O$ amplitude shift at Eirik Drift (using a world ocean surface area of 361.9 × 10^{12} m^2).

The full amplitude shift developed over ~6,000 years. However, it did not develop instaneously: the record shows that it developed in a somewhat sigmoidal manner, and if we approximate this with a linear growth rate, then the sea-level contribution determined from the seawater $\delta^{18}O$ change over the full 6,000 years comes to (0.5 × 6,000 × 1.311 × 10^{-3}) = 3.93 m. Therefore, we find that the median estimate for development of the observed Eirik Drift $\delta^{18}O_{sw}$ shift of –1.3‰ is about 4 m sea-level equivalent of melt-water input from Greenland. Propagation of generous (Gaussian) uncertainties in all parameters in this calculation, using a method similar to Rohling (2000)[Ref. 123], indicates that 1σ = 1.15 m. We conclude that both temporal structure and amplitude of the Eirik Drift sea-water $\delta^{18}O$ record support the Yau et al. (2016) reconstruction of Greenland ice-mass loss[124].

Supplementary Note 4.

Glacio-isostatic assessment of LIG sea-level records

Changes in mass loading at Earth's surface, due to ice-sheet growth and melt and consequent ocean-basin unloading and loading, results in a non-uniform sea-level pattern on a global scale. This is known as glacial isostatic adjustment (GIA). We have previously shown that millennial-scale relative sea level (RSL) fluctuations at Hanish Sill (Red Sea) are proxies for global mean sea-level (GMSL) fluctuations across glacial cycles, although there is a longer-term secular offset between absolute RSL and GMSL values[106,125]. These investigations described an envelope of RSL behaviours at Hanish Sill related to a range of parameters for Earth's viscous response. Other work has demonstrated that modelling of past sea level must account for ice-volume changes both prior to the period of interest, and subsequent to it. To model RSL during the LIG, therefore, at least 3 glacial cycles must be considered prior to the LIG[Ref.126]. The modelling must also consider the impact of different geographical ice-mass distributions, particularly during the preceding penultimate glacial maximum (PGM, marine isotope stage MIS 6)[50,107,126].

To use the continuously sampled Red Sea RSL curves to constrain the volume of polar ice melt during the LIG, we must understand how these RSL curves are affected by GIA. If the GIA signal can be isolated using the models, then it can be removed from the RSL records to recover GMSL. Where that GMSL varies from the present-day 0 m level, the offset may then be interpreted in terms of excess ice-volume melt (or growth). Note, however, that this is complicated by the fact that 'excess ice' will impose a fingerprint of GIA response. To address this, Hay et al. (2014)[Ref.127] sought to highlight those regions where a highstand identified in proxy RSL indicators would overstate GMSL at a given point in time. In their scenario for coincident Greenland and Antarctic melt, Hanish Sill fell outside of these regions. When considering the impact of melt from individual ice sheets, Antarctic melt marginally amplified Red Sea RSL highstands, whereas Greenland melt caused a minor reduction in RSL highstands.

We extend previous GIA modelling to consider:

1) a LIG of ~14 ka duration (130-116 ka) with ice volumes held at present-day values to identify a background GIA signal;
2) four ice scenarios representing variations in both melt volume and geographic ice-mass distribution;
3) a broad suite of Earth models, highlighting results from four models that illustrate the influence of Earth-model choice on reconstructions; and
4) sensitivity tests that—across the above scenarios—analyse the consequences of 'excess ice' reduction or growth on Hanish Sill responses to individual ice-sheet changes.

For the GIA modelling we use a gravitationally self-consistent sea-level theory[128], which accounts for shoreline migration associated with local sea-level variations and changes in the extent of grounded, marine-based ice. The theory incorporates perturbations of Earth's rotation[129] resulting from changing ice-melt or -growth locations. The sea-level equation is solved in an iterative, pseudo-spectral manner[130] with a 1-D spherically symmetric Earth representation. In total, we model responses across a suite of 495 Earth models comprising 3 parameters for lithosphere thickness (71, 96, and 120 km), 11 parameters for upper

mantle viscosity (1×10^{20} to 1×10^{21} Pa s), and 15 parameters for lower mantle viscosity (2×10^{21} to 5×10^{22} Pa s). From these, we highlight four Earth models to display a range of behaviours (*Supplementary Table* 1). Our first three Earth models are similar to those used by Stocchi et al. (2018)[Ref.131]. Our fourth Earth model is chosen to highlight non-standard outlier (<4%) behaviour.

Supplementary Table 1. Earth model parameters used in our glacio-isostatic adjustment modelling.

Earth model	Upper mantle viscosity × 10^{21} Pa s	Lower mantle viscosity × 10^{21} Pa s	Rationale for this Earth model
EM1	1	2	Like VM1
EM2	0.5	5	Like VM2
EM3	0.25	0.1	Lambeck et al. (2014)[Ref.132] (similar to Hay et al. (2014)[Ref.127])
EM4	1	0.5	Extreme outlier for maximum contrast

We investigate RSL behaviour at Hanish Sill during the LIG using the four ice histories developed to investigate sea-level/ice-volume differences between the LGM and PGM[Ref.107]. All four ice histories model ice-volume changes at 2 kyr intervals between 244 ka and present day. Each contains a LIG period between 130 and 116 ka with present-day ice volume. ICE-1 is a version of the ICE-5G ice history[133], and covers two identical glacial cycles. The other three scenarios build on, or adjust, this basic ice history[104]. ICE-2 contains reduced ice volume during the PGM relative to the LGM. ICE-3 also has redistributed ice masses, giving a smaller North American ice sheet, and a larger European ice sheet during the PGM than during the LGM. ICE-4 also has different geographic boundaries for the European ice sheet, after de Boer et al. (2014)[134], while retaining the same ice-volume as ICE-2 and ICE-3.

We find that, when LIG ice volume is held constant, the Hanish Sill RSL response is generally characterised, irrespective of ice history, by an early highstand at the beginning of the interglacial and a subsequent decay toward equilibrium RSL (*Supplementary Figure 5*). For ICE-1 and ICE-2, the initial highstand is higher than GMSL, whereas for ICE-3 and ICE-4 both the initial highstand and the subsequent decay fall below GMSL. Importantly, we note that the total amplitude of this variation across the LIG is only a few metres, and so cannot account for the variations of 10 or more metres observed in our study (Figure 2, main text). For a small outlier subgroup (<4%) of the Earth models investigated, and only for ICE-3 and ICE-4, the viscosity contrast between upper and lower mantle values is such that a highstand is only achieved at the end of the interglacial. We illustrate this outlier behaviour with our fourth Earth model, but note that existing studies focus on EM1-3, as below.

Our main experiments considered only a ‘background’ interglacial scenario with no ice melt or growth greater than present day. To assess sensitivity to ‘excess ice’ variations, we therefore also modelled the responses at Hanish Sill for each individual ice sheet on its own, for individual ice-sheet configurations (*Supplementary Figure 6*). In *Supplementary Figure 6*, configuration A is for an ICE-5G like distribution of ice volume during MIS 6, and configuration B follows an ice-distribution template based on de Boer et al. (2014)[Ref.134]. Responses to Greenland and Antarctic ice-volume changes are similar. The 1:1 line in the graphs indicates no GIA effect, while values below the line indicate that the RSL response is an amplification of the GMSL change (i.e., RSL is higher than GMSL) and values above the line

represent points where RSL is lower than GMSL. For melt associated with the Greenland ice sheet, there is relatively little Earth model sensitivity – the points all cluster tightly along the same line, and generally fall close to the 1:1 line. The impact of Antarctic ice-volume change is highly dependent on the Earth model chosen, but overall such impacts plot along the 1:1 line, which suggests that RSL changes also closely approximate GMSL changes for Antarctic ice-volume changes.

Given that ice scenarios ICE-3 and ICE-4 are considered to be more representative of actual PGM ice-volume distributions[107], and therefore to generate results closer to reality, we infer that the LIG RSL generated for Hanish Sill likely underestimates GMSL by a few metres in absolute terms (slightly more in the later phases than in the beginning). Given this, and the minimal GIA effects that we find at Hanish Sill for Antarctic ice-mass reduction, we consider RSL fluctuations in the first half of the LIG (Figure 2, main text) to be close approximations of GMSL fluctuations. For the second half of the LIG, where ice-mass reduction is considered to have occurred at both Greenland and Antarctica (Figure 3, main text), offsets are again small, and we consider that Red Sea RSL fluctuations again closely approximate GMSL fluctuations.

Where we make GIA corrections to approximately translate Red Sea RSL into GMSL, we use a linear adjustment for the RSL gradient through the LIG from +0 ±0 m at 135 ka, to +4 ±2 m at 115 ka, based on the ICE3 and ICE4 solutions for the three representative EMs 1-3 (*Supplementary Figure 5*). Note that the uncertainties here refer to the gradient through the LIG, not to absolute values.

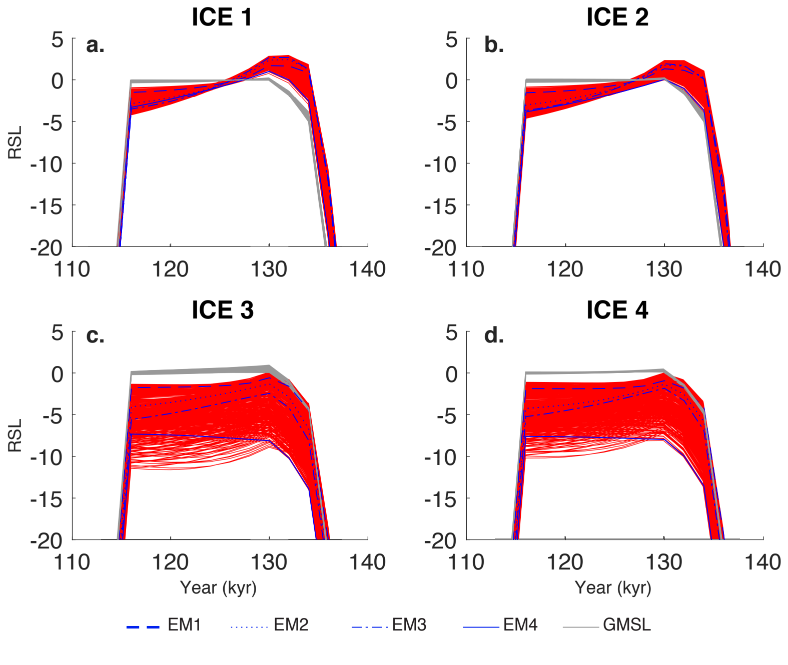

Supplementary Figure 5. Red Sea relative sea level (RSL) *versus* global mean sea level (GMSL) for 495 Earth models at the Hanish Sill, and ice scenarios ICE 1-4. Red lines for all four graphs represent RSL for the full suite of 495 Earth models (EMs) considered. Blue lines represent the four highlighted EMs, and grey lines represent GMSL. **A and B.** For these ice histories RSL tends to overshoot GMSL at the beginning of the interglacial and then decay to an equilibrium value. **C and D.** A greater range in RSL values results from sensitivity to a larger PGM Eurasian ice sheet. Note also the EM sensitivity, where EM1-3 represent the majority in which a highstand occurs at the beginning of the LIG, while EM4 represents an outlier group (<4%) in which the highstand occurs at the end of the LIG.

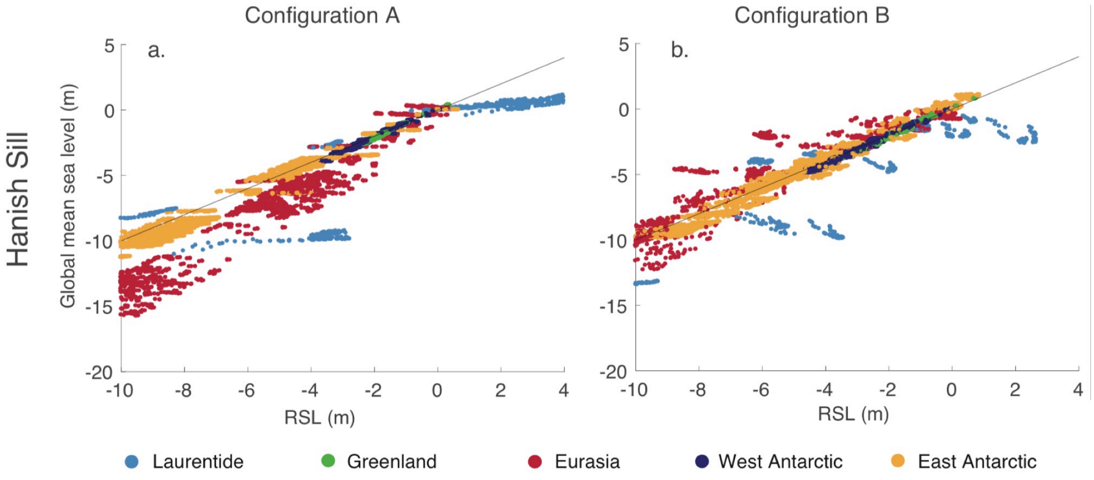

Supplementary Figure 6. Hanish Sill relative sea level (RSL) *versus* global mean sea level (GMSL) for a representative subset of 60 Earth models from our total suite of 495 Earth models. Results are obtained from runs of our GIA model in which individual ice sheets are isolated based on two synthetic ice histories (configuration **A** relies on the ice distribution in ICE-5G, and configuration **B** on that of de Boer et al. (2014)[Ref.134]. The plotted RSL and GMSL signals then represent only the GIA signal associated with the selected individual ice sheet (blue represents North American ice sheets, green represents Greenland, red represents Eurasian ice sheets, and navy and gold represent the West and East Antarctic ice sheets, respectively). Relatively wide horizontal dispersal of red data points indicates considerable sensitivity to EM choice for Eurasian ice sheet responses. In contrast, Antarctic ice-sheet responses (navy and gold) are horizontally tightly clustered, indicating little influence of EM choice. In addition, both Antarctic datapoints (esp. West Antarctica), and Greenland datapoints plot close to the black 1:1 line, which indicates minimal GIA effects at Hanish Sill in response to mass changes in those ice sheets.

Supplementary Note 5.

Consistency between Red Sea and coral-based sea-level reconstructions

Given the dynamic nature of the Red Sea sea-level curve through the LIG, it is difficult to visualise the type of coral record with which this would be consistent. Therefore, we have developed a straightforward model for first-order evaluation. We assume that the Red Sea sea-level record is representative of sea-level movements through the LIG (using the PM solution in this example), following a simple approximate correction for GIA effects (see section 4). For the latter, we use the ICE3 and ICE4 solutions for the three representative EMs, and approximate these by a linear RSL adjustment by +0 m at 135 ka to +4 m at 115 ka, to obtain roughly approximated GMSL values (*Supplementary Figure 7*). These rough adjustments are sufficient because we are concerned with a basic consistency test only.

In essence, we approximate fringing-reef development by assuming total occupation of available accommodation space by reef growth, subject to certain limitations. First, it is assumed that accommodation space for coral growth has an upper depth limit at –1 m water depth, to represent Mean Low Tide over an array of regions, from microtidal regions to regions with large tidal ranges. The chosen value does not affect our conclusions; changes merely shift the simulated reef records up and down in absolute terms. Second, it is assumed that reef-growth rate is optimal over the first 20 m below the upper depth limit, and that it then linearly tapers to zero at about 100 m depth. Third, the model explores two variables:

(a) the influence of reef tolerance to drowning due to rapid sea-level rise; (b) the inverse, namely reef tolerance to rapid sea-level lowering.

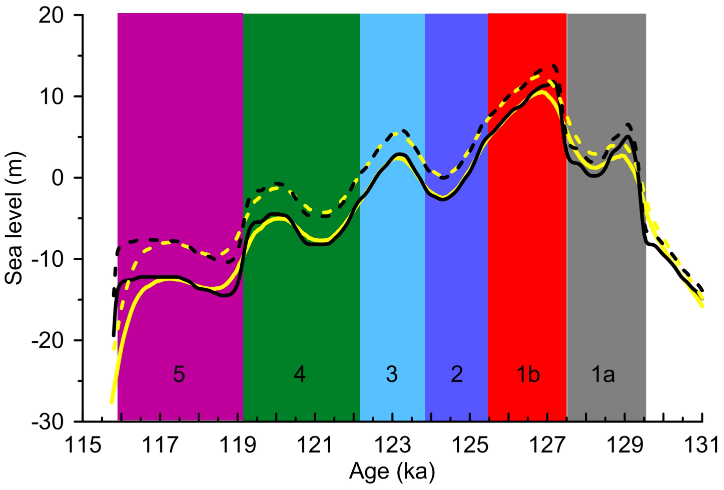

Supplementary Figure 7. Identification of reef-growth phases portrayed in Supplementary Figure 8. Solid lines portray PM (black) and Median (yellow) of the probabilistic Red Sea RSL analysis (Figure 2g). Dashed lines portray PM (black) and Median (yellow) as above, but after schematic GIA correction to rough GMSL values based on linear approximation between 0 m adjustment at 135 ka, and 5 m adjustment at 115 ka. Colours and numbers refer to reef accretion phases in *Supplementary Figure 8*.

The experiments (*Supplementary Figure 8*) start with a sea floor of arbitrary slope. The vertical axis is specified in metres, and the arbitrary slope determines an arbitrary horizontal axis (coastal/shelf width). The chosen slope does not change the modelled pattern of reef formation; it only compresses (steeper slopes) or widens (shallower slopes) the reconstructions laterally. When the simulated sea floor falls within the upper zone of optimal growth, the model allows a reef to fill the entire accommodation space to the limiting depth of –1 m, except when the sea-level lowering or rise thresholds are exceeded, in which case growth is halted. Results over a wide range of specified sea-level lowering threshold values indicate that this parameter has no appreciable impacts so it is ignored hereafter. In contrast, the tolerance threshold value for sea-level rise is critically important.

When sea-level rise exceeds the specified tolerance threshold value, the reef "drowns" and growth is halted in the model until sea-level rise returns below the threshold again. Although coral populations have evolved through the Plio-Pleistocene to cope with rapid sea-level fluctuations[135], threshold values for "keeping up" *versus* "drowning" still vary per taxon. Fast-growing taxa include *Acropora* and *Pocillopora*, while slow-growing corals include *Porites* and faviids[136]. Slow growers have typical growth rates of 10-20 mm/y and fast growers can reach 40-100 mm/y[Refs.136,137]. Individual species growth rates (and hence the dominant assemblages) have an impact on reef accretion rates, and typically reef accretion rates are around 4 mm/y (range: 1-9 mm/y, or 0.4 $^{+0.5}/_{-0.3}$ m per century, m/c)[138,139], with very high values up to 26 mm/y or 2.6 m/c[Ref.138] and only in exceptional cases reaching 30 mm/y or more (3 m/c[Refs.86,140]). Higher values can be accommodated only by landward stepping of reef growth, e.g., the ~5 m/c of melt-water pulse 1a, at around 14.5 ka[Ref.86]. To bracket all options, we explore values from 0.5 m/c to 6 m/c.

The expected reef expression for different modelled LIG phases varies considerably as a function of the specified value of tolerance to "drowning" due to sea-level rise (*Supplementary* Figure 8). It is especially striking that reefs with exceptional drowning tolerances (thresholds ⩾3 m/c; Supplementary Figure 8e-h) are needed to obtain significant expressions of the highest peak (Phase 1b; *Supplementary Figures* 7 and 8). Even higher tolerances (⩾4 m/c; *Supplementary Figure 8f-h*) are needed before that peak would develop

strong expressions. In areas with reef assemblages with tolerances within the observed range (<3m/c), Phase 1b would be hardly developed, if at all (*Supplementary Figure 8a-d*).

Over the range of the most common tolerance levels (0.5 to 2 m/c), the model suggests that most LIG reef deposits should be expected to occur between –2 and +5 m, relative to GMSL, with negligible expression of rapid sea-level variability (*Supplementary Figure 8a-c*), which is reasonably consistent with reported observations (*Supplementary Figure 9*). We, therefore, contend that absence of reef deposits at higher elevations does not imply inconsistency with the Red Sea-based sea-level target curve used here.

Note that our simple exercise reports all results relative to GMSL. It is possible that local GIA and/or tectonic movements relative to GMSL created exceptional "windows" that allowed preservation of Phase 1b expressions even in regions that have reef assemblages with drowning tolerances <3 m/c. Essentially, vertical ground movement (uplift) would in those cases (partially) offset rapid sea-level rise to a sufficient degree to prevent reef drowning. We suggest that more elaborate/realistic predictive modelling along with GIA and tectonic assessment may in future provide clues to identify the most promising (especially uplifting) locations for recovering Phase 1b.

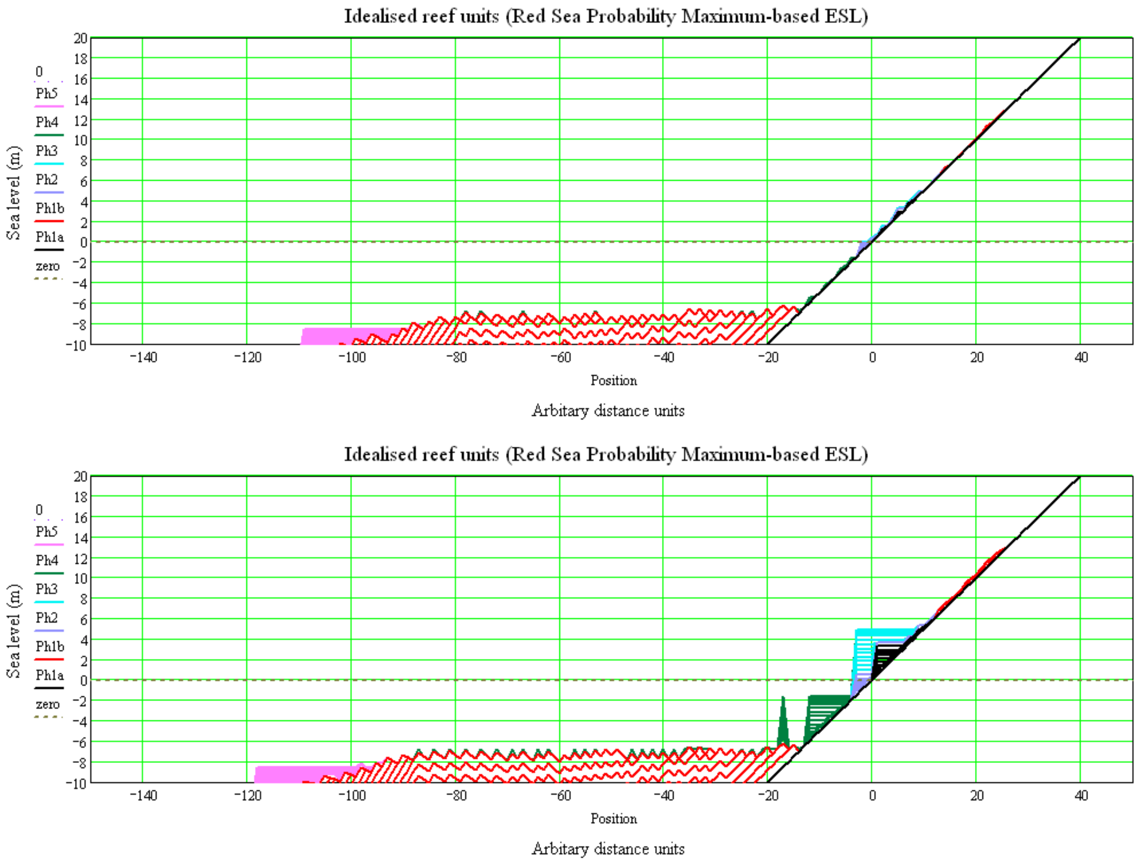

a.

0.5 m/c

b.

1.0 m/c

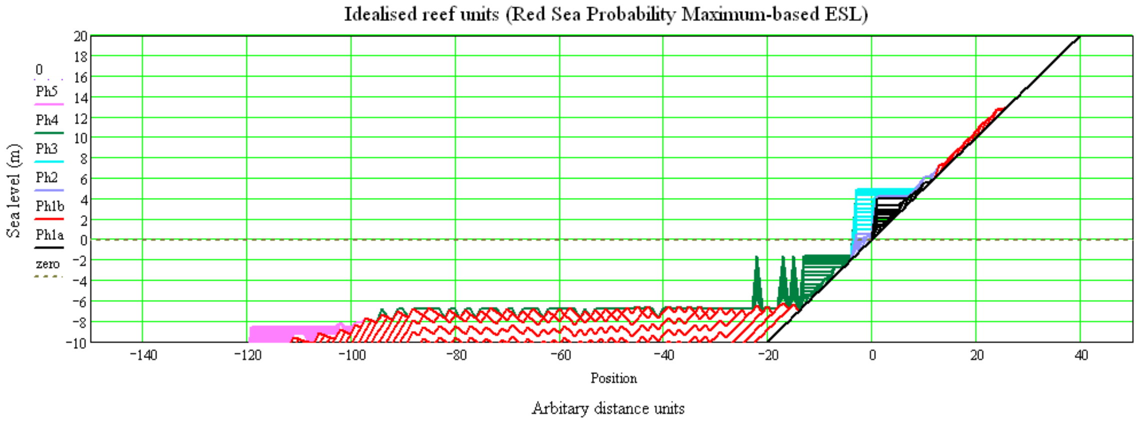

c.

1.5 m/c

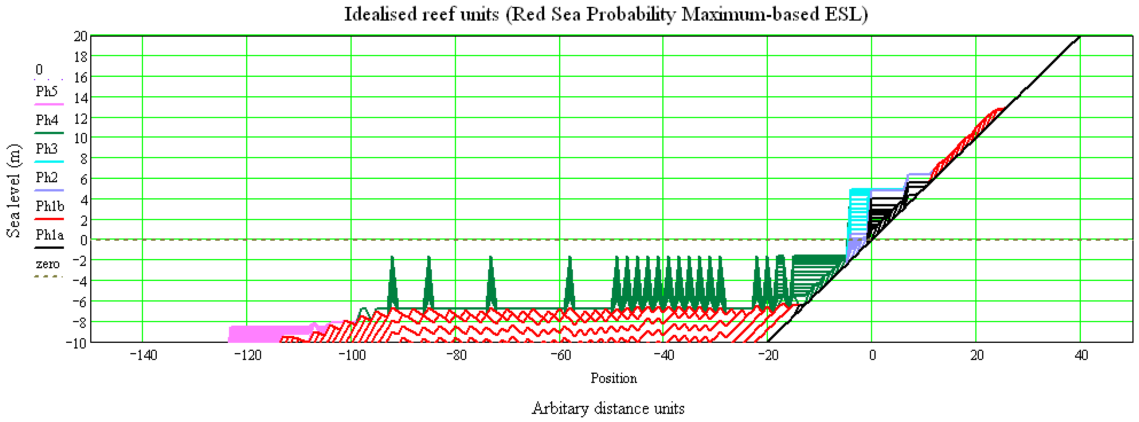

d.

2.0 m/c

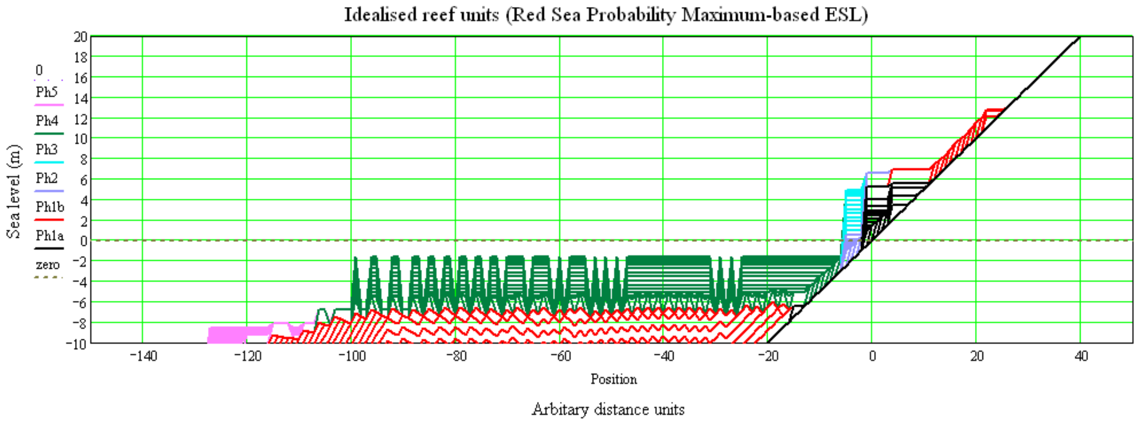

e.

3.0 m/c

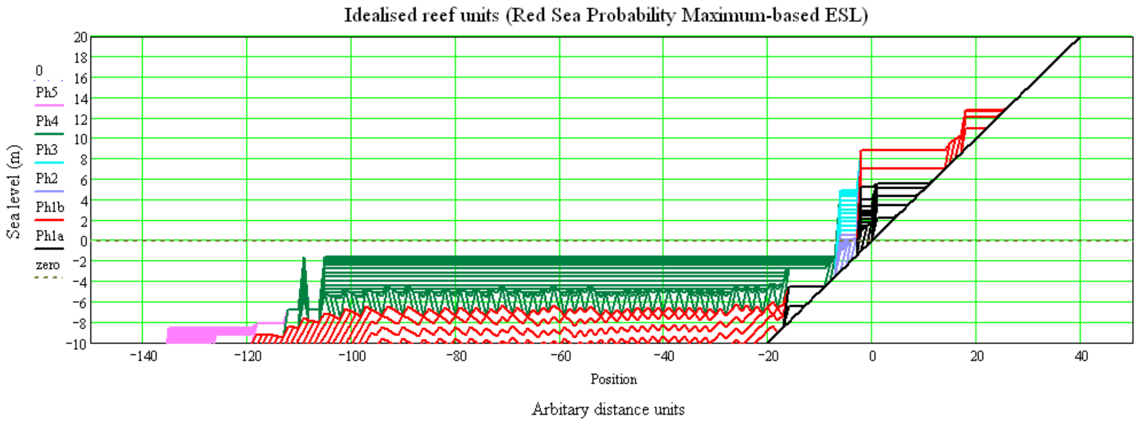

f.

4.0 m/c

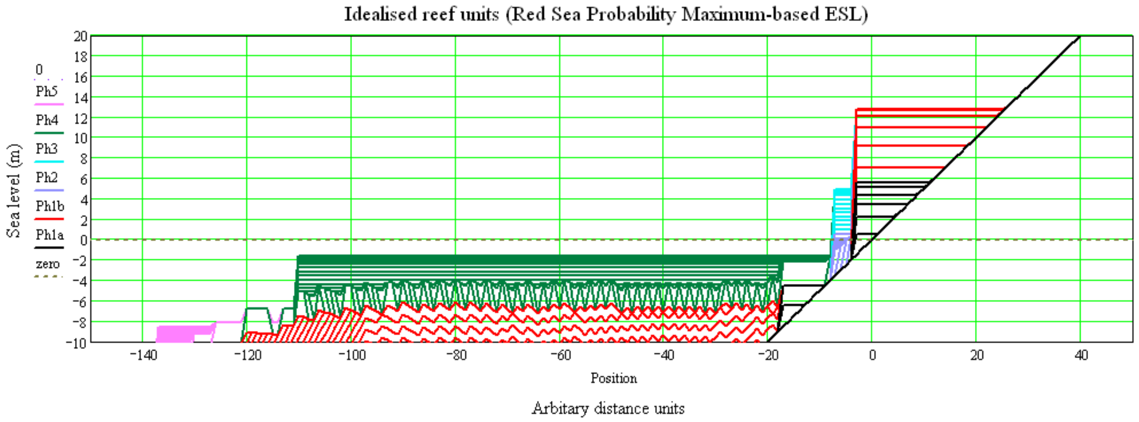

g.

5.0 m/c

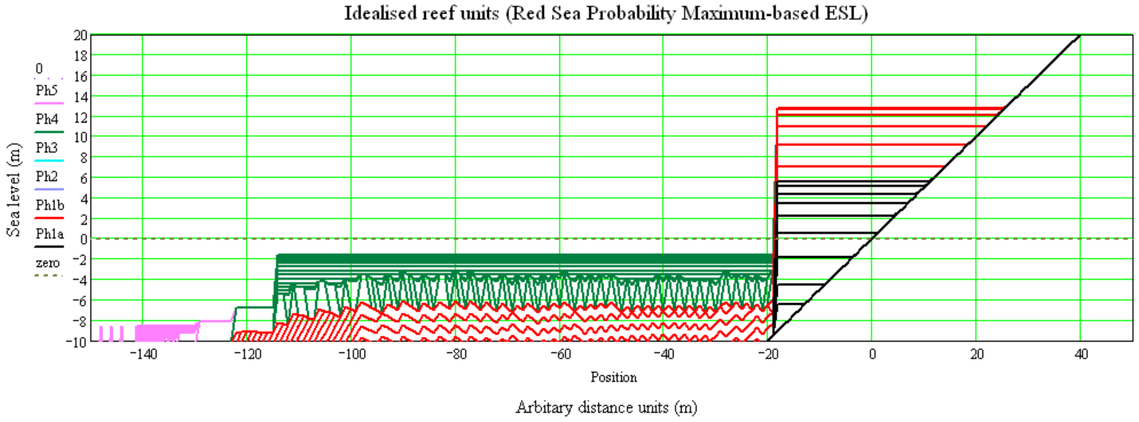

h.

6.0 m/c

Supplementary Figure 8. Idealised modelled reef units for a schematic Red Sea probability maximum-based global mean sea level (GMSL) reconstruction. Successive panels (**a-h**) represent model results for different specified reef "drowning" tolerance threshold values (in terms of rate of sea-level rise), as indicated on the right-hand side. Colours identify different LIG reef phases, as per *Supplementary* Figure 7.

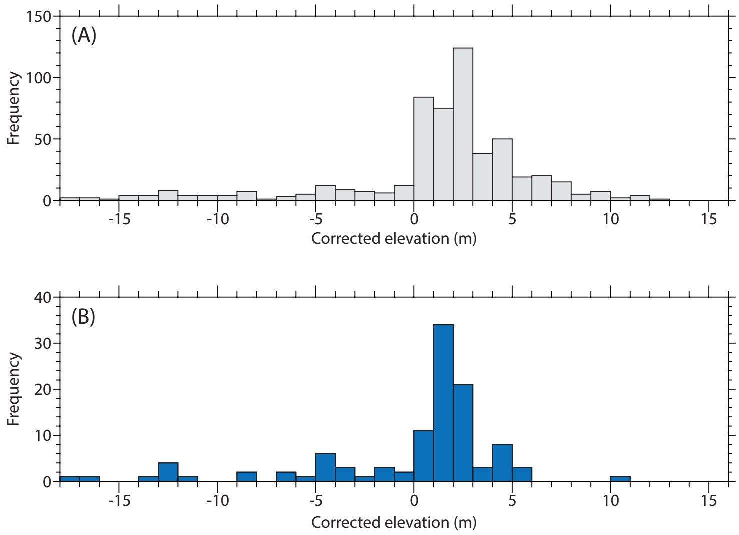

Supplementary Figure 9. Histograms of Last Interglacial coral elevations corrected for tectonic uplift or subsidence since the time of formation (from the compilation of Ref.[87]). **A.** All corals of LIG age[1,8,9,13,17,21,22,26,28-30,37,66–72,76,79,83–90,120–143] (grey). **B.** Subset from A that fulfils age reliability screening criteria (blue) (calcite <2%, ^{232}Th concentration < 2 ppb and $\delta^{234}U_{initial}$ = 147 ± 5 ‰)[8,9,75,79,102,109–114,143,13,147,15,25,29,69–71,73].

Supplementary Note 6.

Rates of sea-level drop between sea-level rise events during the LIG

We acknowledges that it is not yet fully possible to reconcile the high rates of sea-level variability observed in geological archives with current understanding of ice physics. High rates of sea-level rise may be explained through dynamic processes of ice-mass loss that are underestimated[e.g.,167,168]. However, high rates of sea-level lowering require high rates of ice-mass growth, and are less easy to explain. In our Red Sea reconstructions, translated to GMSL as explained in sections 4 and 5, rates of sea-level change are less than –1 m/c for all lowering events bar one (at ~119.1 ka in the $GMSL_{PM}$ reconstruction; i.e., that based on the calculated probability maximum) (*Supplementary Figure 10*). Moreover, average sea-level drop values across entire intervals of sea-level lowering range between –0.23 and –0.63 m/c.

We can consider these values in a rough ball-park assessment. Gross ice accumulation over Greenland and Antarctica is determined by snowfall. This precipitation is not likely to stop because it depends on moisture availability (evaporation) and active weather systems. Gross accumulation over Antarctica today is equivalent to about –0.6 m/c sea-level change[169], and for Greenland about –0.16 m/c sea-level change[170]. Thus, the present sea-level drop at no mass loss would be about –0.76 m/c sea-level change. Yet, the zero mass-loss criterion is unrealistic because melt and calving cannot be expected to be entirely zero. Still the value is considerably larger than the average sea-level drop values we infer across entire intervals of sea-level lowering (–0.23 to –0.63 m/c), while significant warming around Antarctica[171] and reduced sea-ice cover[172] would allow substantially increased moisture supply. This seems to be supported by a 30% accumulation rate increase, from ~30 to ~39 kg m^{-1} y^{-1} at EPICA Dome C[ref.173]. We also note that LIG mass loss is considered to have differed from the present in that fast ice-volume reduction phases led to isostatic rebound with resultant ice-shelf re-grounding, which then may have limited mass loss[174–178].

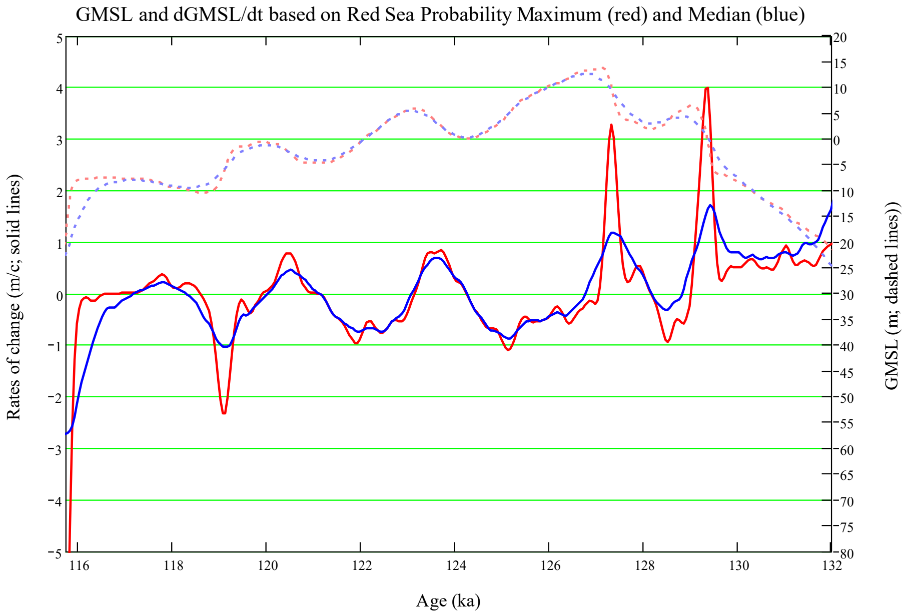

Supplementary Figure 10. Global mean sea level (GMSL, see *Supplementary Figure* 5) based on the Red Sea probability maxima (PM) and median reconstructions (red and blue dashed lines, respectively), along with their rates of change (solid). Data density for KL11 alone is too low <116 ka for robust results.

Supplementary References

1. Walter, R. C. *et al.* Early human occupation of the Red Sea coast of Eritrea during the last interglacial. *Nature* **405**, 65–69 (2000).
2. Bruggemann, H. J. *et al.* Stratigraphy, palaeoenvironments and model for the deposition of the Abdur Reef Limestone: context for an important archaeological site from the last interglacial on the Red Sea coast of Eritrea. *Palaeogeogr. Palaeoclimatol. Palaeoecol.* **203**, 179–206 (2004).
3. Plaziat, J.-C., Reyss, J.-L., Choukri, A. & Cazala, C. Diagenetic rejuvenation of raised coral reefs and precision of dating. The contribution of the Red Sea reefs to the question of reliability of the Uranium-series datings of middle to late Pleistocene key reef-terraces of the world. *Carnets Géologie / Notebooks Geol.* **4**, 2008/04 (2008).
4. Plaziat, J.-C. *et al.* Mise en evidence, sur la cote recifale d'Egypte, d'une regression interrompant brievement le plus haut niveau du dernier interglaciaire (5e); un nouvel indice de variations glacio-eustatiques a haute frequence au Pleistocene? *Bull. la Société Géologique Fr.* **169**, 115–125 (1998).
5. Plaziat, J.-C. *et al.* Quateranry changes in the Egyptian shoreline of the northwestern Red Sea and the Gulf of Suez. *Quat. Int.* **29/30**, 11–22 (1995).
6. Bar, N. *et al.* Last interglacial sea levels and regional tectonics from fossil coral reefs in the northeast Gulf of Aqaba. *Quat. Sci. Rev.* **191**, 41–56 (2018).
7. Yehudai, M. *et al.* U–Th dating of calcite corals from the Gulf of Aqaba. *Geochim. Cosmochim. Acta* **198**, 285–298 (2017).
8. Israelson, C. & Wohlfarth, B. Timing of the Last-Interglacial high sea level on the Seychelles Islands, Indian Ocean. *Quat. Res.* **51**, 306–316 (1999).
9. Dutton, A., Webster, J. M., Zwartz, D., Lambeck, K. & Wohlfarth, B. Tropical tales of polar ice: evidence of Last Interglacial polar ice sheet retreat recorded by fossil reefs of the granitic Seychelles islands. *Quat. Sci. Rev.* **107**, 182–196 (2015).
10. Vyverberg, K. *et al.* Episodic reef growth in the granitic Seychelles during the Last Interglacial: Implications for polar ice sheet dynamics. *Mar. Geol.* **399**, 170–187 (2018).
11. Chappell, J. Geology of coral terraces, Huon Peninsula, New Guinea: A study of Quaternary tectonic movements and sea-Level changes. *Geol. Soc. Am. Bull.* **85**, 553–570 (1974).
12. Chappell, J. & Veeh, H. H. Late Quaternary tectonic movements and sea-level changes at Timor and Atauro Island. *Geol. Soc. Am. Bull.* **89**, 356–368 (1978).
13. Stein, M. *et al.* TIMS U-series dating and stable isotopes of the last interglacial event in Papua New Guinea. *Geochim. Cosmochim. Acta* **57**, 2541–2554 (1993).
14. Aharon, P., Chappell, J. & Compston, W. Stable isotope and sea-level data from New Guinea supports Antarctic ice-surge theory of ice ages. *Nature* **283**, 649–651 (1980).
15. Chen, J. H., Curran, H. A., White, B. & Wasserburg, G. J. Precise chronology of the last interglacial period: ^{234}U-^{230}Th data from fossil coral reefs in the Bahamas. *Geol. Soc. Am. Bull.* **103**, 82–97 (1991).
16. White, B., Curran, H. A. & Wilson, M. A. Bahamian coral reefs yield evidence of a brief sea-level lowstand during the last interglacial. *Carbonates and Evaporites* **13**, 10–22 (1998).
17. Wilson, M. A., Curran, H. A. & White, B. Paleontological evidence of a brief global sea-level event during the last interglacial. *Lethaia* **31**, 241–250 (1998).
18. Hearty, P. J., Hollin, J. T., Neumann, A. C., O'Leary, M. J. & McCulloch, M. T. Global sea-level fluctuations during the Last Interglaciation (MIS 5e). *Quat. Sci. Rev.* **26**, 2090–2112 (2007).
19. Thompson, W. G., Curran, H. A., Wilson, M. A. & White, B. Sea-level oscillations during the last interglacial highstand recorded by Bahamas corals. *Nat. Geosci.* **4**, 684–687 (2011).
20. Skrivanek, A., Li, J. & Dutton, A. Relative sea-level change during the Last Interglacial as recorded in Bahamian fossil reefs. *Quat. Sci. Rev.* **200**, 160–177 (2018).
21. Kerans, C., Zahm, C., Bachtel, S. L., Hearty, P. & Cheng, H. Anatomy of a late Quaternary carbonate island: Constraints on timing and magnitude of sea-level fluctuations, West Caicos, Turks and Caicos Islands, BWI. *Quat. Sci. Rev.* **205**, 193–223 (2019).
22. Neumann, A. C. & Hearty, P. J. Rapid sea-level changes at the close of the last interglacial

(substage 5e) recorded in Bahamian island geology. *Geology* **24**, 775–778 (1996).
23. Hearty, P. J. & Kindler, P. Sea-Level highstand chronology from stable carbonate platforms (Bermuda and the Bahamas). *J. Coast. Res.* **11**, 675–689 (1995).
24. Hearty, P. J. & Neumann, A. C. Rapid sea level and climate change at the close of the Last Interglaciation (MIS 5e): evidence from the Bahama Islands. *Quat. Sci. Rev.* **20**, 1881–1895 (2001).
25. Blanchon, P., Eisenhauer, A., Fietzke, J. & Liebetrau, V. Rapid sea-level rise and reef back-stepping at the close of the last interglacial highstand. *Nature* **458**, 881–884 (2009).
26. Harmon, R. S. *et al.* U-series and amino-acid racemization geochronology of Bermuda: implications for eustatic sea-level fluctuation over the past 250,000 years. *Palaeogeogr. Palaeoclimatol. Palaeoecol.* **44**, 41–70 (1983).
27. Rowe, M. P., Wainer, K. A. I., Bristow, C. S. & Thomas, A. L. Anomalous MIS 7 sea level recorded on Bermuda. *Quat. Sci. Rev.* **90**, 47–59 (2014).
28. Hearty, P. J. & Tormey, B. R. Sea-level change and superstorms; geologic evidence from the last interglacial (MIS 5e) in the Bahamas and Bermuda offers ominous prospects for a warming Earth. *Mar. Geol.* **390**, 347–365 (2017).
29. Muhs, D. R., Simmons, K. R. & Steinke, B. Timing and warmth of the Last Interglacial period: new U-series evidence from Hawaii and Bermuda and a new fossil compilation for North America. *Quat. Sci. Rev.* **21**, 1355–1383 (2002).
30. Hearty, P. J. Revision of the Late Pleistocene stratigraphy of Bermuda. *Sediment. Geol.* **153**, 1–21 (2002).
31. Muhs, D. R., Simmons, K. R., Schumann, R. R. & Halley, R. B. Sea-level history of the past two interglacial periods: new evidence from U-series dating of reef corals from south Florida. *Quat. Sci. Rev.* **30**, 570–590 (2011).
32. Fruijtier, C., Elliott, T. & Schlager, W. Mass-spectrometric ^{234}U-^{230}Th ages from the Key Largo Formation, Florida Keys, United States: Constraints on diagenetic age disturbance. *Geol. Soc. Am. Bull.* **112**, 267–277 (2000).
33. Halley, R. B. & Evans, C. *The Miami Limestone: a guide to selected outcrops and their interpretation*. (1983).
34. Sherman, C. E., Glenn, C. R., Jones, A. T., Burnett, W. C. & Schwarcz, H. P. New evidence for two highstands of the sea during the last interglacial, oxygen isotope substage 5e. *Geology* **21**, 1079–1082 (1993).
35. Ku, T., Kimmel, M. A., Easton, W. H. & O'Neil, T. J. Eustatic Sea Level 120,000 Years Ago on Oahu, Hawaii. *Science (80-.).* **183**, 959–962 (1974).
36. Muhs, D. R. & Szabo, B. J. New uranium-series ages of the Waimanalo Limestone, Oahu, Hawaii: Implications for sea level during the last interglacial period. *Mar. Geol.* **118**, 315–326 (1994).
37. Stearns, H. T. Quaternary shorelines in the Hawai'ian Islands. *B.P. Bish. Museum Bull.* **237**, 1–57 (1978).
38. Hearty, P. J., Kaufman, D. S., Olson, S. L. & James, H. F. Stratigraphy and whole-rock amino acid geochronology of key Holocene and Last Interglacial carbonate deposits in the Hawaiian Islands. *Pacific Sci.* **54**, 423–442 (2000).
39. Szabo, B. J., Ludwig, K. R., Muhs, D. R. & Simmons, K. R. Thorium-230 ages of corals and duration of te Last Interglacial sea-level high stand on Oahu, Hawaii. *Science (80-.).* **266**, 93–96 (1994).
40. Johnson, M. E., Baarli, B. G. & Scott Jr., J. H. Colonization and reef growth on a Late Pleistocene rocky shore and abrasion platform in Western Australia. *Lethaia* **28**, 85–98 (1995).
41. Hearty, P. J. An inventory of last interglacial (*sensu lato*) age deposits from the Mediterranean Basin: a study of isoleucine epimerization and U-series dating. *Zeitschrift für Geomorphol.* **62**, 51–69 (1986).
42. Mauz, B., Fanelli, F., Elmejdoub, N. & Barbieri, R. Coastal response to climate change: Mediterranean shorelines during the Last Interglacial (MIS 5). *Quat. Sci. Rev.* **54**, 89–98 (2012).
43. Zazo, C. *et al.* Pleistocene raised marine terraces of the Spanish Mediterranean and Atlantic

coasts: records of coastal uplift, sea-level highstands and climate changes. *Mar. Geol.* **194**, 103–122 (2003).
44. Carobene, L. Marine notches and sea-cave bioerosional grooves in microtidal areas: examples from the Tyrrhenian and Ligurian Coasts - Italy. *J. Coast. Res.* **31**, 536–556 (2015).
45. Mauz, B. & Antonioli, F. Comment on "Sea level and climate changes during OIS 5e in the Western Mediterranean" by T. Bardají, J.L. Goy, J.L., C. Zazo, C. Hillaire-Marcel, C.J. Dabrio, A. Cabero, B. Ghaleb, P.G. Silva, J. Lario, Geomorphology 104 (2009), 22–37. *Geomorphology* **110**, 227–230 (2009).
46. Bardají, T. *et al.* Sea level and climate changes during OIS 5e in the Western Mediterranean. *Geomorphology* **104**, 22–37 (2009).
47. Ferranti, L. *et al.* Markers of the last interglacial sea-level high stand along the coast of Italy: Tectonic implications. *Quat. Int.* **145–146**, 30–54 (2006).
48. Muhs, D. R., Simmons, K. R., Meco, J. & Porat, N. Uranium-series ages of fossil corals from Mallorca, Spain: The "Neotyrrhenian" high stand of the Mediterranean Sea revisited. *Palaeogeogr. Palaeoclimatol. Palaeoecol.* **438**, 408–424 (2015).
49. Sisma-Ventura, G. *et al.* Last interglacial sea level high-stand deduced from well-preserved abrasive notches exposed on the Galilee coast of northern Israel. *Palaeogeogr. Palaeoclimatol. Palaeoecol.* **470**, 1–10 (2017).
50. Sivan, D. *et al.* Eastern Mediterranean sea levels through the last interglacial from a coastal-marine sequence in northern Israel. *Quat. Sci. Rev.* **145**, 204–225 (2016).
51. Antonioli, F., Ferranti, L. & Kershaw, S. A glacial isostatic adjustment origin for double MIS 5.5 and Holocene marine notches in the coastline of Italy. *Quat. Int.* **145–146**, 19–29 (2006).
52. Jedoui, Y. *et al.* U-series evidence for two high Last Interglacial sea levels in southeastern Tunisia. *Quat. Sci. Rev.* **22**, 343–351 (2003).
53. Mauz, B., Shen, Z., Elmejdoub, N. & Spada, G. No evidence from the eastern Mediterranean for a MIS 5e double peak sea-level highstand. *Quat. Res.* **89**, 505–510 (2018).
54. Chakroun, A., Zaghbib-Turki, D., Miskovsky, J.-C. & Davaud, E. Two Tyrrhenian transgressive cycles in coastal deposits of the Cap Bon Peninsula, Tunisia. *Quaternaire* **20**, 215–226 (2009).
55. Dabrio, C. J. *et al.* Millennial/submillennial-scale sea-level fluctuations in western Mediterranean during the second highstand of MIS 5e. *Quat. Sci. Rev.* **30**, 335–346 (2011).
56. Zazo, C. *et al.* Retracing the Quaternary history of sea-level changes in the Spanish Mediterranean–Atlantic coasts: Geomorphological and sedimentological approach. *Geomorphology* **196**, 36–49 (2013).
57. Hillaire-Marcel, C. *et al.* U-series measurements in Tyrrhenian deposits from Mallorca - Further evidence for two last-interglacial high sea levels in the Balearic Islands. *Quat. Sci. Rev.* **15**, 53–62 (1996).
58. Hillaire-Marcel, C., Carro, O., Causse, C., Goy, J. L. & Zazo, C. Th/U dating of Strombus bubonis-bearing marine terraces in southeastern Spain. *Geology* **14**, 613–616 (1986).
59. Zazo, C. *et al.* The Last Interglacial in the Mediterranean as a model for the present interglacial. *Glob. Planet. Change* **7**, 109–117 (1993).
60. Hearty, P. J., Hollin, J. T. & Dumas, B. Geochronology of Pleistocene littoral deposits on the Alicante and Almeria coasts of Spain. in *Cambios del nivel del mar en Espana en el Cuaternario reciente* **10**, 95–107 (1987).
61. Tuccimei, P. *et al.* Last interglacial sea level changes in Mallorca island (Western Mediterranean). High precision U-series data from phreatic overgrowths on speleothems. *Zeitschrift für Geomorphol.* **50**, 1–21 (2006).
62. Dorale, J. A. *et al.* Sea-Level Highstand 81,000 Years Ago in Mallorca. *Science (80-.).* **327**, 860–863 (2010).
63. Fornós, J. J. *et al.* Phreatic overgrowths on speleothems: a useful tool in structural geology in littoral karstic landscapes. The example of eastern Mallorca (Balearic Islands). *Geodin. Acta* **15**, 113–125 (2002).
64. Tuccimei, P. *et al.* Sea level change at Capo Cacia (NW Sardinia) and Mallorca (Balearic Islands) during oxygen isotope substage 5e, based on Th/U datings of phreatic overgrowths

on speleothems. *Monogr. Nat. Hist. Soc. Balear.* **14**, 121–135 (2007).
65. Muhs, D. R., Meco, J. & Simmons, K. R. Uranium-series ages of corals, sea level history, and palaeozoogeography, Canary Islands, Spain: An exploratory study for two Quaternary interglacial periods. *Palaeogeogr. Palaeoclimatol. Palaeoecol.* **394**, 99–118 (2014).
66. Blanchon, P. Reef demise and back-stepping during the last interglacial, northeast Yucatan. *Coral Reefs* **29**, 481–498 (2010).
67. Blanchon, P. & Shaw, J. Reef drowning during the last deglaciation: Evidence for catastrophic sea-level rise and ice-sheet collapse. *Geology* **23**, 4–8 (1995).
68. Blanchon, P., Jones, B. G. & Ford, D. C. Discovery of a submerged relic reef and shoreline off Grand Cayman: further support for an early Holocene jump in sea level. *Sediment. Geol.* **147**, 253–270 (2002).
69. Zhu, Z. R. *et al.* High-precision U-series dating of Last Interglacial events by mass spectrometry: Houtman Abrolhos Islands, western Australia. *Earth Planet. Sci. Lett.* **118**, 281–293 (1993).
70. Stirling, C. H., Esat, T. M., McCulloch, M. T. & Lambeck, K. High-precision U-series dating of corals from Western Australia and implications for the timing and duration of the Last Interglacial. *Earth Planet. Sci. Lett.* **135**, 115–130 (1995).
71. Stirling, C. H., Esat, T. M., Lambeck, K. & McCulloch, M. T. Timing and duration of the Last Interglacial: evidence for a restricted interval of widespread coral reef growth. *Earth Planet. Sci. Lett.* **160**, 745–762 (1998).
72. O'Leary, M. J., Hearty, P. J. & McCulloch, M. T. Geomorphic evidence of major sea-level fluctuations during marine isotope substage-5e, Cape Cuvier, Western Australia. *Geomorphology* **102**, 595–602 (2008).
73. O'Leary, M. J., Hearty, P. J. & McCulloch, M. T. U-series evidence for widespread reef development in Shark Bay during the last interglacial. *Palaeogeogr. Palaeoclimatol. Palaeoecol.* **259**, 424–435 (2008).
74. O'Leary, M. J. *et al.* Ice sheet collapse following a prolonged period of stable sea level during the last interglacial. *Nat. Geosci.* **6**, 796–800 (2013).
75. Eisenhauer, A., Zhu, Z. R., Collins, L. B., Wyrwoll, K. H. & Eichstätter, R. The Last Interglacial sea level change: new evidence from the Abrolhos islands, West Australia. *Geol. Rundschau* **85**, 606–614 (1996).
76. Whitney, B. B. & Hengesh, J. V. Geomorphological evidence of neotectonic deformation in the Carnarvon Basin, Western Australia. *Geomorphology* **228**, 579–596 (2015).
77. Dumas, B., Hoang, C. T. & Raffy, J. Record of MIS 5 sea-level highstands based on U/Th dated coral terraces of Haiti. *Quat. Int.* **145–146**, 106–118 (2006).
78. Dodge, R. E., Fairbanks, R. G., Benninger, L. K. & Maurrasse, F. Pleistocene sea levels from raised coral reefs of Haiti. *Science (80-.).* **219**, 1423–1425 (1983).
79. Blanchon, P. & Eisenhauer, A. Multi-stage reef development on Barbados during the Last Interglaciation. *Quat. Sci. Rev.* **20**, 1093–1112 (2001).
80. Thompson, W. G. & Goldstein, S. L. Open-system coral ages reveal persistent suborbital sea-level cycles. *Science (80-.).* **308**, 401–404 (2005).
81. Schellmann, G. & Radtke, U. A revised morpho- and chronostratigraphy of the Late and Middle Pleistocene coral reef terraces on Southern Barbados (West Indies). *Earth-Science Rev.* **64**, 157–187 (2004).
82. Scoffin, T. P. *et al.* Calcium carbonate budget of a fringing reef on the west coast of Barbados: Part II - Erosion, sediments and internal structure. *Bull. Mar. Sci.* **302**, 457–508 (1980).
83. Montaggioni, L. F. History of Indo-Pacific coral reef systems since the last glaciation: Development patterns and controlling factors. *Earth-Science Rev.* **71**, 1–75 (2005).
84. Hubbard, D. K. Depth-related and species-related patterns of Holocene reef accretion in the Caribbean and western Atlantic: a critical assessment of existing models. *Annu. Meet. Geol. Soc. Am.* 1–18 (2009). doi:doi: 10.1002/9781444312065.ch1
85. Woodroffe, C. D. & Webster, J. M. Coral reefs and sea-level change. *Mar. Geol.* **352**, 248–267 (2014).
86. Camoin, G. F. & Webster, J. M. Coral reef response to Quaternary sea-level and environmental

changes: State of the science. *Sedimentology* **62**, 401–428 (2015).
87. Hibbert, F. D. *et al.* Coral indicators of past sea-level change: A global repository of U-series dated benchmarks. *Quat. Sci. Rev.* **145**, 1–56 (2016).
88. Scoffin, T. P. Taphonomy of coral reefs: a review. *Coral Reefs* **11**, 57–77 (1992).
89. Perry, C. T. Reef Framework Preservation in Four Contrasting Modern Reef Environments, Discovery Bay, Jamaica. *J. Coast. Res.* **15**, 796–812 (1999).
90. Perry, C. T. Storm-induced coral rubble deposition: Pleistocene records of natural reef disturbance and community response. *Coral Reefs* **20**, 171–183 (2001).
91. Blanchon, P. & Perry, C. T. Taphonomic differentiation of Acropora palmata facies in cores from Campeche Bank Reefs, Gulf of Mexico. *Sedimentology* **51**, 53–76 (2004).
92. Perry, C. T. & Hepburn, L. J. Syn-depositional alteration of coral reef framework through bioerosion, encrustation and cementation: Taphonomic signatures of reef accretion and reef depositional events. *Earth-Science Rev.* **86**, 106–144 (2008).
93. Pandolfi, J. M. & Greenstein, B. J. Taphonomic Alteration of Reef Corals: Effects of Reef Environment and Coral Growth Form. I. The Great Barrier Reef. *Palaios* **12**, 27 (1997).
94. Clark, T. R. *et al.* Testing the precision and accuracy of the U-Th chronometer for dating coral mortality events in the last 100 years. *Quat. Geochronol.* **23**, 35–45 (2014).
95. Blanchon, P., Jones, B. & Kalbfleisch, W. Anatomy of a Fringing Reef Around Grand Cayman: Storm Rubble, Not Coral Framework. *J. Sediment. Res.* **67**, 1–16 (1997).
96. Hubbard, D. K. & Miller, A. I. Production and Cycling of Calcium Carbonate in a Shelf-Edge Reef System (St. Croix, U.S. Virgin Islands): Applications to the Nature of Reef Systems in the Fossil Record. *J. Sediment. Res.* **60**, 335–360 (1990).
97. Edinger, E. N., Burr, G. S., Pandolfi, J. M. & Ortiz, J. C. Age accuracy and resolution of Quaternary corals used as proxies for sea level. *Earth Planet. Sci. Lett.* **253**, 37–49 (2007).
98. Marshall, J. F. & Davies, P. J. Internal structure and Holocene evolution of One Tree Reef, southern Great Barrier Reef. *Coral Reefs* **1**, 21–28 (1982).
99. Hopley, D., Smithers, S. G. & Parnell, K. *The Geomorphology of the Great Barrier Reef: Development, Diversity and Change*. (Cambridge University Press, 2007).
100. Long, A. J. *et al.* Near-field sea-level variability in northwest Europe and ice sheet stability during the last interglacial. *Quat. Sci. Rev.* **126**, 26–40 (2015).
101. Rohling, E. J. *et al.* High rates of sea-level rise during the last interglacial period. *Nat. Geosci.* **1**, 38–42 (2008).
102. Cutler, K. B. *et al.* Rapid sea-level fall and deep-ocean temperature change since the last interglacial period. *Earth Planet. Sci. Lett.* **206**, 253–271 (2003).
103. Moseley, G. E., Smart, P. L., Richards, D. A. & Hoffmann, D. L. Speleothem constraints on marine isotope stage (MIS) 5 relative sea levels, Yucatan Peninsula, Mexico. *J. Quat. Sci.* **28**, 293–300 (2013).
104. Hibbert, F. D. *et al.* Coral indicators of past sea-level change: A global repository of U-series dated benchmarks. *Quat. Sci. Rev.* **145**, 1–56 (2016).
105. Grant, K. M. *et al.* Rapid coupling between ice volume and polar temperature over the past 150,000 years. *Nature* **491**, 744–747 (2012).
106. Grant, K. M. *et al.* Sea-level variability over five glacial cycles. *Nat. Commun.* **5**, 5076 (2014).
107. Rohling, E. J. *et al.* Differences between the last two glacial maxima and implications for ice-sheet, δ18O, and sea-level reconstructions. *Quat. Sci. Rev.* **176**, 1–28 (2017).
108. Cheng, H. *et al.* Improvements in ^{230}Th dating, ^{230}Th and ^{234}U half-life values, and U-Th isotopic measurements by multi-collector inductively coupled plasma mass spectrometry. *Earth Planet. Sci. Lett.* **371–372**, 82–91 (2013).
109. Thompson, W. G., Spiegelman, M. W., Goldstein, S. L. & Speed, R. C. An open-system model for U-series age determinations of fossil corals. *Earth Planet. Sci. Lett.* **210**, 365–381 (2003).
110. Gallup, C. D., Cheng, H., Taylor, F. W. & Edwards, R. L. Direct Determination of the Timing of Sea Level Change During Termination II. *Science (80-.).* **295**, 310–313 (2002).
111. Potter, E.-K. *et al.* Suborbital-period sea-level oscillations during marine isotope substages 5a and 5c. *Earth Planet. Sci. Lett.* **225**, 191–204 (2004).
112. Gallup, C. D., Edwards, R. L. & Johnson, R. G. The Timing of High Sea Levels Over the Past

200,000 Years. *Science (80-.).* **263**, 796–800 (1994).
113. Thomas, A. L. *et al.* Penultimate deglacial sea-level timing from Uranium/Thorium dating of Tahitian corals. *Science (80-.).* **324**, 1186–1189 (2009).
114. Stirling, C. H. *et al.* Orbital Forcing of the Marine Isotope Stage 9 Interglacial. *Science (80-.).* **291**, 290–293 (2001).
115. Vesica, P. L. *et al.* Late Pleistocene Paleoclimates and sea-level change in the Mediterranean as inferred from stable isotope and U-series studies of overgrowths on speleothems, Mallorca, Spain. *Quat. Sci. Rev.* **19**, 865–879 (2000).
116. Marino, G. *et al.* Bipolar seesaw control on last interglacial sea level. *Nature* **522**, 197–201 (2015).
117. Irvali, N. *et al.* Rapid switches in subpolar North Atlantic hydrography and climate during the Last Interglacial (MIS 5e). *Paleoceanography* **27**, PA2207 (2012).
118. Irvali, N. *et al.* Evidence for regional cooling, frontal advances, and East Greenland Ice Sheet changes during the demise of the last interglacial. *Quat. Sci. Rev.* **150**, 184–199 (2016).
119. Cox, K. A. Stable isotopes as tracers for freshwater fluxes into the North Atlantic. (University of Southampton, 2010).
120. Cox, K. A. *et al.* Interannual variability of Arctic sea ice export into the East Greenland Current. *J. Geophys. Res.* **115**, C12063 (2010).
121. Dodd, P. A. *et al.* The freshwater composition of the Fram Strait outflow derived from a decade of tracer measurements. *J. Geophys. Res. Ocean.* **117**, C11005 (2012).
122. Rabe, B. *et al.* Liquid export of Arctic freshwater components through the Fram Strait 1998-2011. *Ocean Sci.* **9**, 91–109 (2013).
123. Rohling, E. J. Paleosalinity: confidence limits and future applications. *Mar. Geol.* **163**, 1–11 (2000).
124. Yau, A. M., Bender, M. L., Robinson, A. & Brook, E. J. Reconstructing the last interglacial at Summit, Greenland: Insights from GISP2. *Proc. Natl. Acad. Sci.* **113**, 9710–9715 (2016).
125. Williams, F. H. A geophysical approach to reconstructing past global mean sea levels using highly resolved sea-level records. (University of Southampton, 2016).
126. Dendy, S., Austermann, J., Creveling, J. R. & Mitrovica, J. X. Sensitivity of Last Interglacial sea-level high stands to ice sheet configuration during Marine Isotope Stage 6. *Quat. Sci. Rev.* **171**, 234–244 (2017).
127. Hay, C. C. *et al.* The sea-level fingerprints of ice-sheet collapse during interglacial periods. *Quat. Sci. Rev.* **87**, 60–69 (2014).
128. Kendall, R. A., Mitrovica, J. X. & Milne, G. A. On post-glacial sea level - II. Numerical formulation and comparative results on spherically symmetric models. *Geophys. J. Int.* **161**, 679–706 (2005).
129. Mitrovica, J. X., Wahr, J., Matsuyama, I. & Paulson, A. The rotational stability of an ice-age earth. *Geophyical J. Int.* **161**, 491–506 (2005).
130. Tamisiea, M. E. Ongoing glacial isostatic contributions to observations of sea level change. *Geophys. J. Int.* **186**, 1036–1044 (2011).
131. Stocchi, P. *et al.* MIS 5e relative sea-level changes in the Mediterranean Sea: Contribution of isostatic disequilibrium. *Quat. Sci. Rev.* **185**, 122–134 (2018).
132. Lambeck, K., Rouby, H., Purcell, A., Sun, Y. & Sambridge, M. Sea level and global ice volumes from the Last Glacial Maximum to the Holocene. *Proc. Natl. Acad. Sci. U. S. A.* **111**, 15296–303 (2014).
133. Peltier, W. R. Global glacial isostasy and the surface of the ice-age Earth: the ICE-5G (VM2) model and GRACE. *Annu. Rev. Earth Sci.* **32**, 111–149 (2004).
134. de Boer, B., Stocchi, P. & van de Wal, R. S. W. A fully coupled 3-D ice-sheet–sea-level model: algorithm and applications. *Geosci. Model Dev.* **7**, 2141–2156 (2014).
135. Mao, Y., Economo, E. P. & Satoh, N. The Roles of Introgression and Climate Change in the Rise to Dominance of Acropora Corals. *Curr. Biol.* (2018). doi:10.1016/j.cub.2018.08.061
136. Riegl, B., Berumen, M. & Bruckner, A. Coral population trajectories, increased disturbance and management intervention: a sensitivity analysis. *Ecol. Evol.* **3**, 1050–1064 (2013).
137. Dullo, W.-C. Coral growth and reef growth: a brief review. *Facies* **51**, 33–48 (2005).

138. Morgan, K. M., Perry, C. T., Smithers, S. G., Johnson, J. A. & Gulliver, P. Transitions in coral reef accretion rates linked to intrinsic ecological shifts on turbid-zone nearshore reefs. *Geology* **44**, 995–998 (2016).
139. Perry, C. T. & Morgan, K. M. Bleaching drives collapse in reef carbonate budgets and reef growth potential on southern Maldives reefs. *Sci. Rep.* **7**, 40581 (2017).
140. Perry, C. T., Smithers, S. G., Gulliver, P. & Browne, N. K. Evidence of very rapid reef accretion and reef growth under high turbidity and terrigenous sedimentation. *Geology* **40**, 719–722 (2012).
141. Camoin, G. F., Ebren, P., Eisenhauer, A., Bard, E. & Faure, G. A 300 000-yr coral reef record of sea level changes, Mururoa atoll (Tuamotu archipelago, French Polynesia). *Palaeogeogr. Palaeoclimatol. Palaeoecol.* **175**, 325–341 (2001).
142. Edwards, R. L., Cheng, H., Murrell, M. T. & Goldstein, S. J. Protactinium-231 dating of carbonates by thermal ionization mass\rspectrometry: Implications for Quaternary climate change. *Science (80-.).* **276**, 782–786 (1997).
143. Muhs, D. R., Pandolfi, J. M., Simmons, K. R. & Schumann, R. R. Sea-level history of past interglacial periods from uranium-series dating of corals, Curaçao, Leeward Antilles islands. *Quat. Res.* **78**, 157–169 (2012).
144. Multer, H. G., Gischler, E., Lundberg, J., Simmons, K. R. & Shinn, E. A. Key Largo Limestone revisited: Pleistocene shelf-edge facies, Florida Keys, USA. *Facies* **46**, 229–271 (2002).
145. Speed, R. C. & Cheng, H. Evolution of marine terraces and sea level in the last interglacial, Cave Hill, Barbados. *Geol. Soc. Am. Bull.* **116**, 219–232 (2004).
146. Toscano, M. A. & Lundberg, J. Early Holocene sea-level record from submerged fossil reefs on the southeast Florida margin. *Geology* **26**, 255–258 (1998).
147. Toscano, M. A., Macintyre, I. G. & Lundberg, J. Last interglacial reef limestones, northeastern St. Croix, US Virgin Islands-evidence of tectonic tilting and subsidence since MIS 5.5. *Coral Reefs* **31**, 27–38 (2012).
148. Andersen, M. B. *et al.* The timing of sea-level high-stands during Marine Isotope Stages 7.5 and 9: Constraints from the uranium-series dating of fossil corals from Henderson Island. *Geochim. Cosmochim. Acta* **74**, 3598–3620 (2010).
149. Andersen, M. B. *et al.* High-precision U-series measurements of more than 500,000 year old fossil corals. *Earth Planet. Sci. Lett.* **265**, 229–245 (2008).
150. Bard, E. *et al.* Pleistocene sea levels and tectonic uplift based on dating of corals from Sumba Island, Indonesia. *Geophys. Res. Lett.* **23**, 1473–1476 (1996).
151. Bard, E., Hamelin, B., Fairbanks, R. G. & Zindler, A. Calibration of the ^{14}C timescale over the past 30,000 years using mass spectrometric U–Th ages from Barbados corals. *Nature* **345**, 405–410 (1990).
152. Coyne, M. K., Jones, B. & Ford, D. Highstands during Marine Isotope Stage 5: evidence from the Ironshore Formation of Grand Cayman, British West Indies. *Quat. Sci. Rev.* **26**, 536–559 (2007).
153. Dia, A. ., Cohen, A. ., O'Nions, R. . & Jackson, J. . Rates of uplift investigated through ^{230}Th dating in the Gulf of Corinth (Greece). *Chem. Geol.* **138**, 171–184 (1997).
154. Edwards, R. L., Chen, J. H., Ku, T.-L. & Wasserburg, G. J. Precise Timing of the Last Interglacial Period from Mass Spectrometric Determination of Thorium-230 in Corals. *Science (80-.).* **236**, 1547–1553 (1987).
155. Esat, T. M., McCulloch, M. T., Chappell, J., Pillans, B. & Omura, A. Rapid Fluctuations in Sea Level Recorded at Huon Peninsula During the Penultimate Deglaciation. *Science (80-.).* **283**, 197–201 (1999).
156. Hamelin, B., Bard, E., Zindler, A. & Fairbanks, R. G. ^{234}U/^{238}U mass spectrometry of corals: How accurate is the UTh age of the last interglacial period? *Earth Planet. Sci. Lett.* **106**, 169–180 (1991).
157. Ludwig, K. R., Muhs, D. R., Simmons, K. R., Halley, R. B. & Shinn, E. A. Sea-level records at ~ 80 ka from tectonically stable platforms: Florida and Bermuda. *Geology* **24**, 211 (1996).
158. McCulloch, M. T. & Mortimer, G. E. Applications of the ^{238}U– ^{230}Th decay series to dating of fossil and modern corals using MC-ICPMS. *Aust. J. Earth Sci.* **55**, 955–965 (2008).

159. Muhs, D. R., Simmons, K. R., Kennedy, G. L. & Rockwell, T. K. The last interglacial period on the Pacific Coast of North America: Timing and paleoclimate. *Geol. Soc. Am. Bull.* **114**, 569–592 (2002).
160. Muhs, D. R., Simmons, K. R., Kennedy, G. L., Ludwig, K. R. & Groves, L. T. A cool eastern Pacific Ocean at the close of the Last Interglacial complex. *Quat. Sci. Rev.* **25**, 235–262 (2006).
161. Muhs, D. R. *et al.* Sea-level history during the Last Interglacial complex on San Nicolas Island, California: implications for glacial isostatic adjustment processes, paleozoogeography and tectonics. *Quat. Sci. Rev.* **37**, 1–25 (2012).
162. Stirling, C. H. High-precision U-series dating of corals from Western Australia: implications for last interglacial sea levels. (The Australian National University, 1996).
163. Frank, N. *et al.* Open system U-series ages of corals from a subsiding reef in New Caledonia: Implications for sea level changes, and subsidence rate. *Earth Planet. Sci. Lett.* **249**, 274–289 (2006).
164. McMurtry, G. M., Campbell, J. F., Fryer, G. J. & Fietzke, J. Uplift of Oahu, Hawaii, during the past 500 k.y. as recorded by elevated reef deposits. *Geology* **38**, 27–30 (2010).
165. Zazo, C. *et al.* Quaternary marine terraces on Sal Island (Cape Verde archipelago). *Quat. Sci. Rev.* **26**, 876–893 (2007).
166. Vezina, J., Jones, B. & Ford, D. Sea-level highstands over the last 500,000 years; evidence from the Ironshore Formation on Grand Cayman, British West Indies. *J. Sediment. Res.* **69**, 317–327 (1999).
167. Alley, R. B. *et al.* Oceanic Forcing of Ice-Sheet Retreat: West Antarctica and More. *Annu. Rev. Earth Planet. Sci.* **43**, 207–231 (2015).
168. Bamber, J. L., Oppenheimer, M., Kopp, R. E., Aspinall, W. P. & Cooke, R. M. Ice sheet contributions to future sea-level rise from structured expert judgment. *Proc. Natl. Acad. Sci.* 201817205 (2019). doi:10.1073/pnas.1817205116
169. Cazenave, A. *et al.* Global sea-level budget 1993–present. *Earth Syst. Sci. Data* **10**, 1551–1590 (2018).
170. van den Broeke, M. R. *et al.* On the recent contribution of the Greenland ice sheet to sea level change. *Cryosph.* **10**, 1933–1946 (2016).
171. Capron, E. *et al.* Temporal and spatial structure of multi-millennial temperature changes at high latitudes during the Last Interglacial. *Quat. Sci. Rev.* **103**, 116–133 (2014).
172. Wolff, E. W. *et al.* Southern Ocean sea-ice extent, productivity and iron flux over the past eight glacial cycles. *Nature* **440**, 491–496 (2006).
173. Wolff, E. W. *et al.* Changes in environment over the last 800,000 years from chemical analysis of the EPICA Dome C ice core. *Quat. Sci. Rev.* **29**, 285–295 (2010).
174. Bradley, S. L., Hindmarsh, R. C. A., Whitehouse, P. L., Bentley, M. J. & King, M. A. Low post-glacial rebound rates in the Weddell Sea due to Late Holocene ice-sheet readvance. *Earth Planet. Sci. Lett.* **413**, 79–89 (2015).
175. Gomez, N., Pollard, D. & Mitrovica, J. X. A 3-D coupled ice sheet – sea level model applied to Antarctica through the last 40 ky. *Earth Planet. Sci. Lett.* **384**, 88–99 (2013).
176. Gomez, N., Pollard, D. & Holland, D. Sea-level feedback lowers projections of future Antarctic Ice-Sheet mass loss. *Nat. Commun.* **6**, 8798 (2015).
177. Konrad, H., Sasgen, I., Pollard, D. & Klemann, V. Potential of the solid-Earth response for limiting long-term West Antarctic Ice Sheet retreat in a warming climate. *Earth Planet. Sci. Lett.* **432**, 254–264 (2015).
178. Kingslake, J. *et al.* Extensive retreat and re-advance of the West Antarctic Ice Sheet during the Holocene. *Nature* **558**, 430–434 (2018).
